# Pairs of Adjacent Conserved Noncoding Elements Separated by Conserved Genomic Distances Act as *Cis*-Regulatory Units

**DOI:** 10.1093/gbe/evy196

**Published:** 2018-09-03

**Authors:** Lifei Li, Nicolai K H Barth, Eva Hirth, Leila Taher

**Affiliations:** Division of Bioinformatics, Department of Biology, Friedrich-Alexander-Universität Erlangen-Nürnberg, Erlangen, Germany

**Keywords:** conserved noncoding elements (CNEs), genome architecture, *cis*-regulatory elements, transposable elements, epistasis

## Abstract

Comparative genomic studies have identified thousands of conserved noncoding elements (CNEs) in the mammalian genome, many of which have been reported to exert *cis*-regulatory activity. We analyzed ∼5,500 pairs of adjacent CNEs in the human genome and found that despite divergence at the nucleotide sequence level, the inter-CNE distances of the pairs are under strong evolutionary constraint, with inter-CNE sequences featuring significantly lower transposon densities than expected. Further, we show that different degrees of conservation of the inter-CNE distance are associated with distinct *cis*-regulatory functions at the CNEs. Specifically, the CNEs in pairs with conserved and mildly contracted inter-CNE sequences are the most likely to represent active or poised enhancers. In contrast, CNEs in pairs with extremely contracted or expanded inter-CNE sequences are associated with no *cis*-regulatory activity. Furthermore, we observed that functional CNEs in a pair have very similar epigenetic profiles, hinting at a functional relationship between them. Taken together, our results support the existence of epistatic interactions between adjacent CNEs that are distance-sensitive and disrupted by transposon insertions and deletions, and contribute to our understanding of the selective forces acting on *cis*-regulatory elements, which are crucial for elucidating the molecular mechanisms underlying adaptive evolution and human genetic diseases.

## Introduction

Over 97% of the human genome does not code for proteins, but instead is mainly involved in gene expression regulation and chromosome structure maintenance (Koop and Hood 1994; Lander et al. 2001). The relevance of noncoding sequences is strengthened by the observation that variants in these regions are often associated with complex diseases (Pennisi 2012). In particular, 5% of the genome is under evolutionary constraint, but only 40% of this 5% consists of protein-coding genes (Lindblad-Toh et al. 2011). Although sequence conservation between two or more species can be the result of either functional constraint or insufficient divergence time, conserved noncoding sequences are of particular interest for their potential as *cis*-regulatory elements (Duret and Bucher 1997; Hardison et al. 1997; Frazer 2001; Pennacchio and Rubin 2001). *Cis*-regulatory elements are DNA sequences containing binding sites for transcription factors (TFs) whose binding is needed to activate, sustain or repress transcription (Ong and Corces 2011). Promoters and enhancers are the best understood types of *cis*-regulatory elements. While promoters are proximal to their target genes, residing immediately upstream of their transcription start sites (TSSs), enhancers and other distal *cis*-regulatory elements such as silencers and insulators can be located at any distance from the gene(s) they regulate (Kolovos et al. 2012; Stadhouders et al. 2012). Long-distance interactions between proximal and distal *cis*-regulatory elements by means of chromatin loops are central to the currently accepted model of transcriptional regulation, and find support in a growing body of evidence (Kadauke and Blobel 2009). The mechanisms by which these chromatin loops are established and maintained and their effects on transcription have just begun to be elucidated. Thus, it has been shown that the protein complexes, mediator and cohesin, have especially important roles in bringing into and maintaining the TFs at the enhancers and silencers and the basic transcriptional machinery at promoters in physical proximity (Andersson et al. 2015). Despite this progress in our understanding of their mechanisms of action and their well-established roles in organismal development and human disease (Stathopoulos and Levine 2005; Bhatia and Kleinjan 2014), the identification of *cis*-regulatory elements remains a major challenge. In the past decade, large international efforts such as the Encyclopedia of DNA Elements (ENCODE) Project (ENCODE Project Consortium 2012) have extensively used technologies such as chromatin immunoprecipitation followed by next-generation sequencing (ChIP-seq), DNase-seq and chromosome conformation capture to create genome-wide maps of *cis*-regulatory elements. However, distal *cis*-regulatory elements are thought to be mostly cell-type specific, and an exhaustive experimental identification of *cis*-regulatory elements in all cells and under all conditions is likely to be infeasible. The development of computational approaches attempting to predict functions beyond the available data and helping to decipher the principles of transcriptional regulation is crucial.

Multispecies alignments of genomic sequences have identified thousands of discrete evolutionary conserved sequences in the human genome (Bejerano 2004; Sandelin et al. 2004; Woolfe et al. 2004; Siepel 2005), which are commonly referred to as conserved noncoding elements (CNEs). Remarkably, the distance between two such CNEs, in particular, “ultraconserved” (Bejerano 2004; Sandelin et al. 2004) and “highly conserved” noncoding elements (Woolfe et al. 2004), has also been shown to be conserved (Sun et al. 2006, 2009), suggesting that the sequences in between CNEs, hereinafter referred to as inter-CNE sequences, contain additional functional elements or/and are structurally relevant. In addition, Sun et al. (2006, 2009) reported that the transposon densities of the inter-CNE sequences appear to be correlated with the evolutionary conservation of their lengths, with longer sequences exhibiting higher transposon densities. Indeed, the distribution of transposable elements is not uniform across the vertebrate genome. To further investigate the conservation of inter-CNE sequences and the impact of transposon activity in the functions associated with the corresponding CNEs, we constructed and analyzed a data set of 5,657 pairs of adjacent CNEs in the human genome that are widely conserved in mammals. We observed that pairs of adjacent CNEs for which the inter-CNE distance is either conserved or shorter than in the common mammalian ancestor comprise lower densities of DNA transposons, long terminal repeats (LTRs), long interspersed nuclear elements (LINEs), and short interspersed nuclear elements (SINEs) than expected by chance. Furthermore, in agreement with the aforementioned literature, we found that pairs of CNEs for which the inter-CNE distance is longer compared with the common mammalian ancestor comprise greater transposon densities. Moreover, our findings indicate that while shorter inter-CNE sequences are associated with transposon losses, longer inter-CNE sequences are associated with transposon gains, in particular of SINEs, indicating an active role for transposons in the contraction or expansion of inter-CNE sequences in the mammalian genome. To investigate the possible consequences of inter-CNE contractions and expansions, we quantified the *cis*-regulatory activities of the CNEs and their inter-CNE sequences as a function of the evolutionary conservation of the corresponding inter-CNE distances. Specifically, we found that CNEs of pairs with conserved or mildly contracted inter-CNE sequences are more likely to be poised or active enhancers than CNEs in pairs with extremely contracted or expanded inter-CNE sequences. Moreover, we observed that often the two CNEs of a pair, rather than only one, are associated with *cis*-regulatory activity, at a rate that is significantly higher than what is expected by chance. Thus, we hypothesize that CNEs in adjacent pairs are functionally related and that extreme changes in the inter-CNE distance alter the interaction between them, weakening or disrupting their *cis*-regulatory activity. Consistently, transposon activity in the human genome would often mediate the evolution of novel functions by fostering or disrupting interactions between adjacent *cis*-regulatory-active CNEs. Our study provides novel insights into the complex interactions that occur between CNEs in the human genome and can be used to identify candidates for epistatic interactions underlying disease penetrance and severity.

## Materials and Methods

### Data Sets of Conserved Elements

PhastCons elements conserved across 46 placental mammals were downloaded for the hg19 human genome assembly from the UCSC genome browser (http://hgdownload.soe.ucsc.edu/goldenPath/hg19/database/phastConsElements46wayPlacental.txt.gz, last accessed September 12, 2018; Siepel 2005). This data set contains 3,743,478 conserved elements whose definition is based on a multiple sequence alignment (Siepel 2005). Adjacent conserved elements with a distance smaller than 100 bp were merged together. Resulting conserved elements shorter than 100 bp were filtered out. The final data set contained 529,143 conserved elements.

PhastCons elements conserved across 60 placental mammals were downloaded for the mm10 mouse genome assembly from the UCSC genome browser (http://hgdownload.soe.ucsc.edu/goldenPath/mm10/database/phastConsElements60wayPlacental.txt.gz, last accessed September 12, 2018). The original data set contained 5,256,349 elements. After processing the data set in the same way as above, there were 649,917 elements left.

### Genomic Annotation

Human and mouse gene annotations were obtained from GENCODE (version 19 and M12, respectively; Harrow et al. 2012) in the GTF format. We filtered the transcripts of genes labeled as protein coding. The processed human and mouse data sets (see Data Sets of Conserved Elements) were mapped to the annotation file and classified into four categories: Exons, untranslated regions (UTRs), introns, and intergenic regions, respectively. If an element overlapped with multiple categories, only the category with the highest precedence was assigned, with the following, descending order: Exon, UTR, intron, and intergenic region. Due to our selection of only protein-coding genes for the categorization, we also refer to the exon category as “protein coding.”

### Orthologous Elements

Reference genome assemblies for the following 23 species were downloaded from the UCSC genome browser (Kent et al. 2002; http://hgdownload.cse.ucsc.edu/goldenPath/, last accessed September 12, 2018): hg19 (human), mm10 (mouse), rn6 (rat), panTro4 (chimp), calJac3 (marmoset), canFam3 (dog), dasNov3 (armadillo), loxAfr3 (elephant), monDom5 (opossum), ornAna1 (platypus), galGal4 (chicken), taeGut2 (zebra finch), chrPic1 (turtle), anoCar2 (lizard), xenTro3 (frog), danRer10 (zebrafish), fr3 (fugu), rheMac8 (rhesus macaque), myoLuc2 (microbat), ailMel1 (panda), susScr3 (pig), turTru2 (dolphin), and cerSim1 (rhinoceros).

The human and mouse genomes were used as reference. We identified orthologous elements as reciprocal best hits (RBHs) using BLAT (Kent 2002) with default parameters. Among the multiple resulting alignments per sequence, we kept the best hit only. In a second alignment, the orthologous sequences detected by the first alignment were aligned back against to the human genome for all 22 species. If the best hit of the second alignment overlapped with the original human sequence, we considered both sequences as true orthologs. RBHs are a common proxy for orthology. Although BLAST is the software most usually used for identifying such hits, the number and size of the genomes involved in the study posed a computational limitation. BLAT’s running time is a fraction of the time required to run BLAST.

### Distances between Pairs of Elements in the Common Mammalian Ancestor

The ancestral character state of the distance between pairs of conserved elements was reconstructed using the FastAnc and contMap functions in the R package phytools (version 0.5-64; Revell 2012). The function contMap maps a continuous trait onto a phylogenetic tree using the function fastAnc. The function fastAnc estimates the states at internal nodes of the phylogenetic tree based on maximum likelihood methods and interpolating the states along each edge using equation (2) of Felsenstein (1985). Branch lengths represent times of species divergence and were obtained from TimeTree (http://www.timetree.org/, last accessed September 12, 2018; Kumar and Hedges 2011). The taxonomy tree for the 23 species mentioned above was generated with the phyloT online tool (phylot.biobyte.de) with a random breakdown of polytomies. Estimates for the common mammalian ancestor were based on inferences for the node indicated with a star (see supplementary fig. S1, Supplementary Material online). The inference of the character state at any given node requires the states at its direct child nodes. Thus, we could only reconstruct the distance between pairs of conserved elements in the common mammalian ancestor for pairs that were conserved in platypus.

### Relative Genome Size-Normalized Distance Difference between CNE–CNE Pairs

In order to identify evolutionary distance changes of the sequences between the CNEs in the CNE–CNE pairs, we calculated the relative distance difference (nRDD):

$$\text{nRDD}=\frac{\frac{d_{h}}{G_{h}}-\frac{d_{r}}{G_{r}}}{\frac{\frac{d_{h}}{G_{h}}+\frac{d_{r}}{G_{r}}}{2}},$$

where *d*_h_ and *d*_r_ were the distances between the midpoints of two CNEs in a human (or mouse) pair and their two orthologous CNEs in the common mammalian ancestor, respectively, and *G*_h_ and *G*_r_ the genome sizes of human (mouse) and the common mammalian ancestor, respectively (see supplementary table S1, Supplementary Material online). The estimated genome size of the common mammalian ancestor is 3,270,000,000 bp (Organ et al. 2011).

### nRDD Groups

CNE–CNE data sets were divided into ten equal-sized groups based on their nRDD values, from low to high. Specifically, group 1 comprised the 10% of the data set with the lowest nRDD values (extreme contractions compared with the common mammalian ancestor), group 10 the 10% of the data set with the highest nRDD values (extreme expansions compared with the common mammalian ancestor), and group 5 the 10% with median nRDD values (most conserved compared with the common mammalian ancestor), with the nRDD values of the remaining groups accordingly in between.

### CNE Conservation

Sequence conservation of CNEs was evaluated using PhastCons scores (http://hgdownload.soe.ucsc.edu/goldenPath/hg19/database/phastCons100way.txt.gz, last accessed September 12, 2018; Siepel 2005). For each conserved element in pair of an nRDD group we computed the mean PhastCons score. Differences between groups were tested for statistical significance using Wilcoxon rank-sum test.

### Transposon Density

For each inter-CNE sequence in a CNE–CNE pair, we randomly sampled a genomic sequence of the same length. Then, we computed the fraction of base pairs of the inter-CNE sequences and their random counterparts overlapping with LTR, DNA, LINE, and SINE transposons. Finally, we compared the fractions obtained for all inter-CNE sequences with those of their random counterparts using a Wilcoxon signed-rank test. We repeated this procedure 1,000 times. The empirical *P*-value reported is the proportion of tests with a *P*-value smaller than or equal to 0.05. Transposon annotation was obtained from the latest human and mouse repeat databases (version 4.05) that were downloaded from the RepeatMasker website (http://www.repeatmasker.org/, last accessed September 12, 2018).

### Relative Transposon Difference (nRTD)

To quantify evolutionary changes in the inter-CNE sequences associated with transposon insertions and/or deletions, we introduced the relative transposon difference (nRTD):

$$\text{nRTD}=\frac{\frac{T_{h}}{G_{h}}-\frac{T_{r}}{G_{r}}}{\frac{\frac{T_{h}}{G_{h}}+\frac{T_{r}}{G_{r}}}{2}},$$

where *T*_h_ is the length of the inter-CNE sequence (considered as the sequence in between the midpoints of the two CNEs of a CNE–CNE pair) annotated as transposon (see Transposon Density) in human (or mouse), *T*_r_ is the corresponding length for the orthologous inter-CNE sequence in the common mammalian ancestor, and *G*_h_ and *G*_r_ are the sizes of the human (mouse) and the common mammalian ancestor genomes, respectively. Similar metrics have been proposed and implemented by other authors (Sun et al. 2006; Babarinde and Saitou 2016).

### Distance to the Nearest TSS

Distance to the nearest TSS for every CNE–CNE pair was based on annotation from GENCODE (see Genomic Annotation) and with respect to midpoints of the complete sequences of the CNE–CNE pairs.

### Epigenetic Profiles

We examined the enrichment levels (fold enrichments compared with the control experiment, which represents the background signal) for H4K27ac, H3K27me3, H3K4me1, H3K4me3, and H3K9me3 quantified by ChIP sequencing (ChIP-seq) in 28 different human tissues by the Roadmap Epigenomics Project (http://www.roadmapepigenomics.org/, last accessed September 12, 2018; Bernstein 2010) and in 58 different mouse tissues by the ENCODE Mouse Project (http://www.mouseencode.org/, last accessed September 12, 2018; Stamatoyannopoulos et al. 2012). In particular, for each CNE–CNE pair in the human genome we computed the mean enrichment levels for each histone modification and each tissue across: 1) two 300-bp-long sequences, each centered at each of the CNEs, 2) the inter-CNE sequence, and 3) the 200-bp-long regions within the inter-CNE sequence with the highest mean enrichment levels for the histone modification under consideration.

### Enrichment Levels of the Inter-CNE Sequence Compared with the Genome-Wide Expectation

For each histone modification and inter-CNE sequence, we identified the 200-bp-long region within the inter-CNE sequence with the highest mean enrichment level. Next, we randomly sampled a genomic sequence with the same length as the inter-CNE sequence and identified the 200-bp-long region with the highest enrichment level for the histone modification under consideration. Finally, we compared the enrichment levels obtained for the inter-CNE sequences with those of their random counterparts using a Wilcoxon rank-sum test. The random sampling procedure and subsequent comparison were performed for each histone modification separately 1,000 times. The empirical *P*-value reported is the proportion of tests with a *P*-value smaller than or equal to 0.05.

### Self-Organizing Maps

In order to cluster histone modification states (H3K27me3, H3K27ac, H3K4me1, H3K4me3, and H3K9me3) in CNEs and the inter-CNE sequences of CNE–CNE pairs, we applied Kohonen self-organizing maps (SOMs; Kohonen 1990).

An SOM is a clustering and visualization algorithm that maps similar input vectors onto contiguous locations of a discrete low-dimensional grid (the map) in a topology-preserving manner. The SOM is a two-dimensional array of units represented by a codebook vector $m_{i}$ that has the same dimension as the vectors in the input data set. The units are connected to adjacent units by a neighborhood function which is a decreasing function of the training iteration t and dictates the topology of the map. We used a hexagonal topology with *n =* 12 units and initialized the codebook vectors to random vectors in the input data set. In each training step *t*, a vector *x*(*t*) is drawn randomly from the input data set and the Euclidean distance is calculated between the input data sample and all the codebook vectors. The best-matching unit is chosen to be that represented by the codebook vector with greatest similarity with the input vector. After finding the best-matching unit, the codebooks $m_{1},m_{2},\ldots ,m_{n}$ are updated as follows:

$$m_{i}(t+1)=m_{i}(t)+h_{ci}(t)(x(t)-m_{i}(t)),$$

where $h_{ci}(t)$ is the neighborhood function around the nearest codebook vector $m_{c}$. In particular, we used the “bubble” neighborhood function, which is constant over the whole neighborhood of the nearest codebook and zero elsewhere. We trained the SOM over 1,000 iterations.

CNE–CNE pairs were clustered according to their mean enrichment levels for the aforementioned five different histone modifications across the 21 human (58 mouse) tissues for which data were available: 1) The 300-bp-long sequences centered at the center of the left and 2) right CNEs of each pair; 3) the inter-CNE sequences of the pair; and 4) the 200-bp-long region within the inter-CNE sequence of the pair with the highest mean enrichment level for each of the histone modifications. For each CNE–CNE pair, the two 300-bp-long sequences centered at the center of the left and right CNEs were sorted according to their enrichment levels for H3K27ac across 21 tissues. Specifically, the CNE with the highest overall enrichment level was labeled as “CNE_h” and the other CNE was labeled as “CNE_l.” There was a total of 420 (21 tissues × 4 sequences × 5 histone modifications) human (1,160 mouse) features for each CNE–CNE pair. The kohonen R package (Wehrens and Buydens 2007) was used for constructing and visualizing the SOMs.

### Enrichments of CNE–CNE Groups in SOM Units

Enrichments were reported as odds ratios, and their significance was evaluated using single-sided Fisher’s exact tests. *P*-values were adjusted for multiple testing using the false discovery rate (FDR). For SOM unit *j* and group of CNE–CNE pairs *i* (where a group is defined as either an nRDD group or a group of human and mouse orthologous CNE–CNE pairs, see below), the number of CNE–CNE pairs in *i* associated with unit *j* was compared with the number of CNE–CNE pairs in other groups. The odds ratio was calculated as follows:

$$\text{odds}–\text{ratio}=\frac{\frac{N_{ij}}{N_{i\bar{j}}}}{\frac{N_{i\bar{j}}}{N_{i\bar{j}}}},$$

where $N_{ij}$ is the number of CNE–CNE pairs in group *i* that are associated with unit *j*, $N_{i\bar{j}}$ is the number of CNE–CNE pairs in group *i* that are not associated with unit *j*, $N_{i\bar{j}}$ is the number of CNE–CNE pairs that are not in group *i* but are associated with unit *j*, and $N_{i\bar{j}}$ is the number of CNE–CNE pairs that are neither in group *i* nor associated with unit *j*.

### Clusters of SOM Units

Human and mouse SOMs are based on the same histone modifications but on different tissues, depending on their availability. To identify equivalent units between the human and mouse SOMs we computed for each unit the mean enrichment level across all tissues for each histone modification at each CNE, at the inter-CNE sequences, and the 200-bp-long regions with the highest enrichment levels within the latter. Based on the means, we computed the reciprocally most similar (in Euclidean distance sense) pairs of human and mouse units. Thus, we found that human SOM units 1, 3, 4, 5, 7, 9, 10, 11 and 12 are reciprocally most similar to mouse SOM units 5, 10, 12, 9, 11, 1, 2, 7 and 4, respectively. Clusters of mouse SOM units were defined by grouping together the reciprocally most similar mouse SOM unit to each human SOM unit in each of the four clusters. Mouse SOM units 3, 6, and 8 have no unambiguous equivalent among the human SOM units (see supplementary fig. S2*a*, Supplementary Material online). Hierarchical clustering (complete linkage based on the Euclidean distance, supplementary fig. S2*b*, Supplementary Material online) suggests that mouse SOM units 3 and 8 are closest to units 4 and 7, while unit 6 is closest to unit 2. (Hence, units 3 and 8 were assigned to the cluster containing 4 and 7 (iv), while unit 6 was assigned to the cluster containing 2 (i).)

### Functional Analysis

We tested various data sets, each consisting of ten groups, for enrichment of gene functions. To do so, we compared the set of closest genes of both CNEs in a CNE–CNE pair for all pairs in a group to background genes (closest genes of all CNE–CNE pairs). The Database for Annotation, Visualization and Integrated Discovery (DAVID Bioinformatics Resources 6.8, http://david.abcc.ncifcrf.gov/, last accessed September 12, 2018; Huang et al. 2009a, 2009b) was applied to test for functional and pathway statistical enrichment analysis. In particular, we focused on the categories: UP_KEYWORDS and KEGG_PATHWAY and identified enriched categories based on an FDR corrected *P*-value.

### Human and Mouse Orthologous CNE–CNE Pairs

In order to compare expansions and contractions relative to the common mammalian ancestor in the human and mouse genomes, we identified orthologous adjacent CNE–CNE pairs in both species (“AO CNE–CNE pairs”). Specifically, for each CNE–CNE pair in the human data set (see Data Sets of Conserved Elements and Results), we verified that: 1) each of the CNEs has an ortholog in the mouse genome (see Orthologous Elements), and 2) the CNE orthologs are adjacent in the mouse genome, that is, there is no conserved element between the two CNEs in the mouse data set (see Data Sets of Conserved Elements). For those CNE orthologs fulfilling the two conditions, we further verified that they had been identified as a CNE–CNE pair in the mouse genome (see Results). Analogously, we defined a set of AO CNE–CNE pairs starting from the CNE–CNE pairs in the mouse data set. Human and mouse data sets of CNE–CNE pairs rely on phastCons elements conserved across different species (see Data Sets of Conserved Elements). Hence, the two data sets of AO CNE–CNE pairs are different. Out of the 5,657 CNE–CNE pairs in the human genome, 1,466 pairs have adjacent orthologs for both CNEs in the mouse genome; analogously, 1,436 out of the 2,084 CNE–CNE pairs in the mouse genome have adjacent orthologs for both CNEs in the human genome. The intersection of human and mouse mammalian conserved CNE–CNE pairs contains 1,411 AO CNE–CNE pairs and these are the AO CNE–CNE pairs that were used for the analyses. Furthermore, we separated the CNE–CNE pairs into nine categories, based on the relative conservation of their inter-CNE sequences compared with the common mammalian ancestor in the human and mouse genomes: 1) “H1-3; M1-3” (nRDD groups 1–3 in human, and 1–3 in mouse), 2) “H1-3; M4-7” (nRDD groups 1–3 in human, and 4–7 in mouse), 3) “H1-3; M8-10” (nRDD groups 1–3 in human, and 8–10 in mouse), 4) “H4-7; M1-3” (nRDD groups 4–7 in human, and 1–3 in mouse), 5) “H4-7; M4-7” (nRDD groups 4–7 in human, and 4–7 in mouse), 6) “H4-7; M8-10” (nRDD groups 4–7 in human, and 8–10 in mouse), 7) “H8-10; M1-3” (nRDD groups 8–10 in human, and 1–3 in mouse), 8) “H8-10; M4-7” (nRDD groups 8–10 in human, and 4–7 in mouse), and 9) “H8-10; M8-10” (nRDD groups 8–10 in human, and 8–10 in mouse).

## Results

### Inter-CNE Distances Are Highly Conserved in Mammals and Vertebrates

**Fig. 1.**
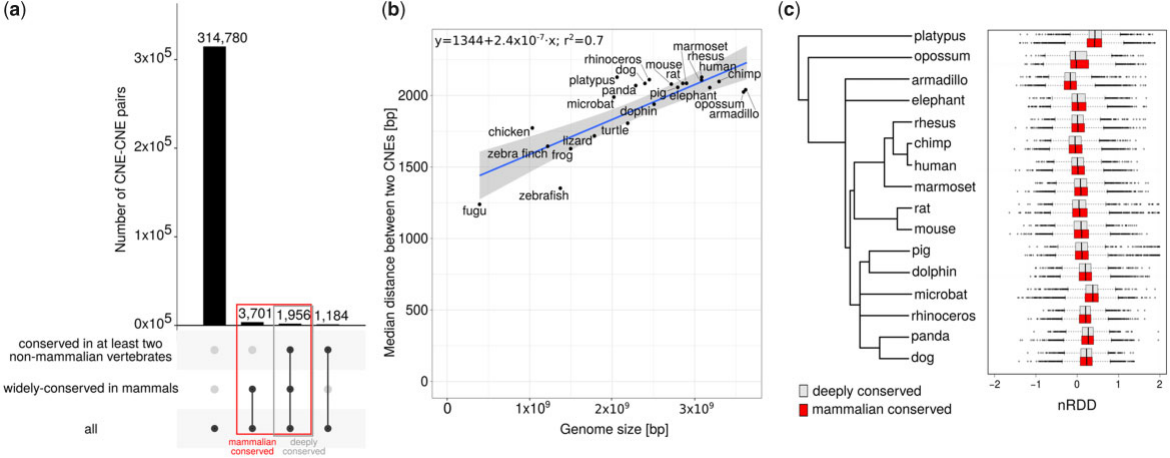
—Distances between CNEs in CNE–CNE pairs in the human genome are highly conserved. (*a*) We identified 321,621 pairs of adjacent CNEs or “CNE–CNE pairs” in the human genome. A total of 5,657 CNE–CNE pairs were conserved in at least human and platypus (“mammalian conserved,” in red); of these CNE–CNE pairs, 1,956 were also conserved in at least two nonmammalian vertebrates (“deeply conserved,” in gray). In total, 3,140 CNE–CNE pairs were conserved in at least human and two nonmammalian vertebrates (but not necessarily in platypus). The relationships between these data sets are shown as disjoint sets using UpSet plots (Lex et al. 2014), which were generated with the UpSetR package (Lex et al. 2014). (*b*) The median distance between two adjacent CNEs (*y* axis) for 23 species against the genome size (*x* axis), with linear regression (blue line), confidence interval (shaded area), and Pearson’s *r*^2^ shown. (*c*) Phylogenic tree for 16 mammalian species (left) and distribution of nRDD values for each species (right, gray: mammalian conserved CNE–CNE pairs, red: deeply conserved CNE–CNE pairs).

We surveyed a total of 529,143 conserved elements from the PhastCons database (Siepel 2005) in the human genome, of which 144,153 overlapped protein-coding sequences and 384,990 did not (see Materials and Methods). We further refer to the latter as CNEs. In particular, 21,367 CNEs were located within UTRs, 158,398 within introns, and 205,225 within intergenic regions. To assess the evolutionary conservation of the genomic distance between conserved elements we first identified pairs of adjacent conserved elements, defined as two conserved elements with no conserved elements in between. Pairs of adjacent conserved elements involving at least one element longer than 1,000 bp were excluded from additional analyses to minimize artifacts, because large sequences are likely to comprise multiple conserved elements. Next, we searched for the ortholog of each element in other 22 vertebrate species (see Materials and Methods). Pairs of adjacent elements with orthologs located at more than 250,000 bp from each other in the genome of any of the 23 vertebrate species involved in the study were also discarded to avoid spurious inferences derived from incorrect orthology assignment and large rearrangement events. As a result, we obtained 321,621 pairs of adjacent CNEs in the human genome, with a median distance of 3,081 bp between them (see supplementary fig. S3, Supplementary Material online). We dubbed these pairs of adjacent CNEs CNE–CNE pairs. CNE–CNE pairs displayed a variable level of conservation. Specifically, 5,657 CNE–CNE pairs were widely conserved in mammals (see Materials and Methods and supplementary table S2, Supplementary Material online) and 3,140 were conserved in at least two nonmammalian vertebrates among lizard, turtle, chicken, zebra finch, frog, fugu, and zebrafish (see fig. 1*a*). Furthermore, many of the CNE–CNE pairs that were widely conserved in mammals were further conserved outside mammals in other vertebrates, with 1,956 also being conserved in at least two nonmammalian vertebrates (see fig. 1*a*). We further refer to the CNE–CNE pairs that are widely conserved in mammals as “mammalian conserved” and to those that are conserved in additional nonmammalian vertebrates as “deeply conserved.” Deeply conserved CNE–CNE pairs spanned shorter genomic distances than their mammalian counterparts (medians 1,733 and 2,127 bp, respectively, *P*-value = 0.005, Wilcoxon rank-sum test).

We next used the CNE–CNE pairs in the human genome and their orthologs in the 16 mammalian species included in the study to infer the distance between the corresponding orthologs in the common mammalian ancestor (see Materials and Methods). We found that the distance between CNEs in CNE–CNE pairs, or inter-CNE distances, was shorter in the human compared with the common mammalian ancestor’s genome (medians 2,127 vs. 2,384 bp and 1,733 vs. 1,964 bp for mammalian and deeply conserved CNE–CNE pairs, respectively, both *P*-values < 0.01, Wilcoxon signed-rank test). In addition, for all mammalian and deeply conserved human CNE–CNE, pairs, the distances between the elements in the pairs were strongly positively correlated with those in the common mammalian ancestor (Spearman’s rho ≥ 0.9). We made similar observations for the remaining 15 mammalian species included in the study, indicating that the distances between adjacent conserved elements are generally well conserved. Consistent with pervasive sequence accumulation in larger genomes, the median distances between the CNEs in the pairs were strongly and positively correlated with the genome size (Pearson’s *r* = 0.8, *P*-value = 1.4 × 10^−6^, see fig. 1*b*). Thus, to evaluate changes in the distances between the CNEs in the pairs in modern mammalian genomes with respect to the common mammalian ancestor that do not simply reflect changes in their overall genome sizes, we devised a genome size-normalized distance difference relative to the common mammalian ancestor (nRDD, see supplementary fig. S4, Supplementary Material online, and Materials and Methods). More precisely, we first subtracted the genome size-normalized distance in the common mammalian ancestor from that in the modern species of interest, and then divided this difference by the mean of the two genome size-normalized distances. The difference between the genome size-normalized distances varies between −1 and 1, but does not reflect the percent change, making it difficult to compare across inter-CNE sequences and to identify extreme changes. As an illustration, change in a genome size-normalized distance from 0.1 to 0.2 (100% increase) is presumably more relevant than a change from 0.2 to 0.3 (50% increase). To capture the relative differences, we divided by the mean. nRDD values lie between −2 and 2. Specifically, nRDD values close to zero indicate no changes in the distances with respect to the common mammalian ancestor; positive nRDD values represent expansions and negative nRDD values contractions in the species of interest compared with the common mammalian ancestor. We found that the distributions of nRDD values for the 5,657 mammalian conserved CNE–CNE pairs were centered close to zero, with a few exceptions (see fig. 1*c*). In addition, nRDD values were only weakly positively correlated with the distances between the pairs of elements. Thus, the Spearman’s rho correlation coefficient for CNE–CNE pairs in the human genome was 0.31 (both *P*-values < 2.2 × 10^−16^, see supplementary fig. S5, Supplementary Material online). This finding makes it evident that the nRDD and distance measure different aspects of genome evolution and advocating for the use of the nRDD as a valuable metric to capture contractions and expansions of the genome. The deeply conserved data sets exhibited similar trends.

### Changes in the Inter-CNE Distance Are Associated with Transposon Activity

**Fig. 2.**
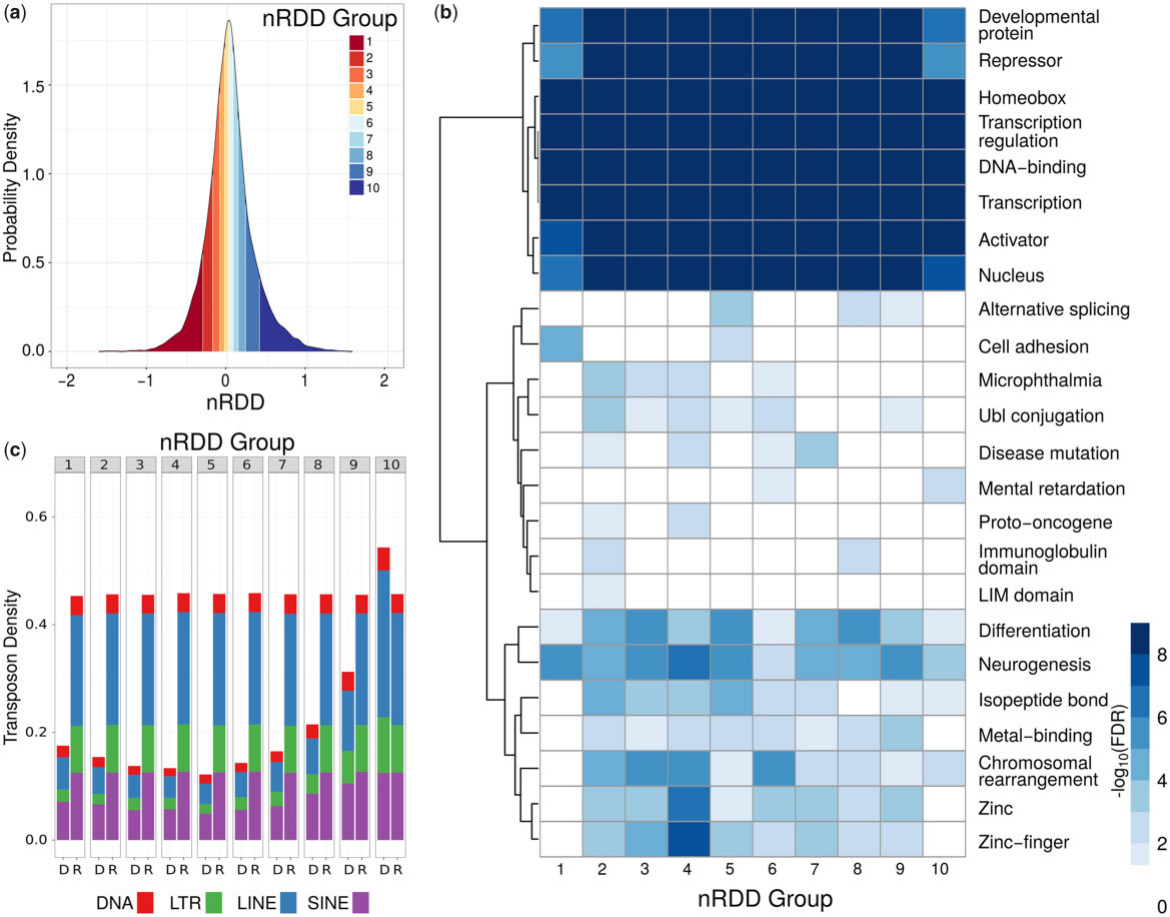
—Sequence properties for mammalian conserved CNE–CNE pairs in the human genome in different nRDD groups. (*a*) Distribution of nRDD values for mammalian conserved CNE–CNE pairs in the human genome. Ten equal-sized groups (deciles) defined according to increasing nRDD values are denoted with different colors. (*b*) For any given square, the color illustrates the significance (−log_10_(FDR)) of the association between each group (*x* axis) and functional annotation, computed based on the annotation of the nearest gene to each CNE–CNE pair. For ease of visualization, FDR ≥ 0.05 are represented in white. (*c*) Mean transposon densities for the four main transposon families (DNA: LTRs, LINEs, and SINEs) for the inter-CNE sequences of the CNE–CNE pairs in each nRDD group (“D”), compared with those of random genomic sequences comprising the same distances (“R”). Except for group 10, inter-CNE sequences of CNE–CNE pairs in all groups CNE–CNE exhibited significantly lower transposon densities than expected.

In order to understand the factors associated with changes in the inter-CNE distances in the human genome, we separated the CNE–CNE pairs into ten equal-sized groups (deciles) defined by increasing nRDD values (or nRDD groups, see fig. 2*a* and Materials and Methods). Thus, the CNE–CNE pairs exhibiting extreme changes in their inter-CNE distances relative to the common mammalian ancestor are in nRDD groups 1–3 (contracted inter-CNE sequences) and 8–10 (expanded inter-CNE sequences), while groups 4–7 contain CNE–CNE pairs with inter-CNE distances changing at the expected pace. Despite the differences in the conservation of the inter-CNE distance among the nRDD groups, the CNEs themselves did not show any significant variation in sequence conservation (see Materials and Methods). The genes in the neighborhood of the CNE–CNE pairs in each group were associated with multiple biological processes, molecular functions, and protein domains (see fig. 2*b* and Materials and Methods). In general, groups corresponding to low nRDD values (contracted inter-CNE distances) were associated with larger number of biological processes, molecular functions, and protein domains than those corresponding to high nRDD values (expansions).

Notably, we found that the mean DNA, LTR, LINE, and SINE transposon densities of the inter-CNE sequences increased regularly with the nRDD value (see fig. 2*c*). In addition, with the exception of group 10, all groups exhibited significantly lower mean transposon densities than expected by chance (from 0.12 to 0.31, with a mean of 0.17, compared with the random expectation of 0.46, empirical *P*-values < 0.05, see Materials and Methods and fig. 2*c*). In contrast, the mean transposon density for group 10 was similar to that of the human genome. Moreover, also the transposon composition of group 10 was similar to that of the human genome, with LINEs being the most abundant transposons (mean density = 0.27) followed by SINEs (mean density = 0.12), while other groups exhibited approximately the same relative abundances of LINEs and SINEs (both with mean densities from 0.04 to 0.11, see fig. 2*c*). Deeply conserved CNE–CNE pairs showed similar trends, although their mean densities were generally slightly lower (see supplementary fig. S6, Supplementary Material online).

To determine whether the observed differences in transposon densities between CNE–CNE pairs in distinct nRDD groups are due to relatively recent events, we examined the transposon annotation for the inter-CNE sequences of the CNE–CNE pairs in the human genome and their orthologs in the 16 mammalian species included in the study, and used phylogenetic analysis to infer the transposon densities in the common mammalian ancestor (see Materials and Methods). Thus, we found 1,714 inter-CNE sequences comprising transposons in the common mammalian ancestor but not in human, which can be interpreted as likely transposon losses (see supplementary fig. S7, Supplementary Material online). The proportion of human inter-CNE sequences exhibiting such transposon losses systematically decreased from nRDD group 1 to nRDD group 10 for all transposon classes. In contrast, the proportion of human inter-CNE sequences with transposon insertions increased. In particular, 90% of the inter-CNE sequences in group 10 exhibited insertions comprised SINEs. Moreover, independently of the group, most insertions with respect to the common mammalian ancestor correspond to SINEs, consistent with their role in shaping the structure of the mammalian genome (Richardson et al. 2015). Interestingly, all groups displayed evidence of both deletions and insertions of all transposon classes, with the balance shifted toward the former or the latter depending on the group. Thus, on average, inter-CNE sequences in group 1 appear to have experienced deletions, while inter-CNE sequences in group 10 appear to have experienced insertions. In addition, we observed 567 inter-CNE sequences that did not comprise any transposons of any class in either human or the common mammalian ancestor. Transposon-free regions have been suggested to represent extended regulatory regions or serve as spacers between regulatory sequences (Simons et al. 2005). Further, we found that the proportion of such sequences was relatively high for groups featuring conserved inter-CNE distances (nRDD groups 4–7) and low for groups representing extreme contractions or expansions (nRDD groups 1–3 and 8–10, respectively), consistent with an evolutionary constraint on their lengths. Together, these results strongly support an active role for transposon activity in the contraction and expansion of inter-CNE sequences.

### Different Conservation Levels of Inter-CNE Distances Are Associated with Distinct Epigenetic Profiles

Next, we investigated the epigenetic profiles of mammalian and deeply conserved CNE–CNE pairs. While high enrichment levels for histone modifications H3K4me1 and H3K27ac are associated with active enhancers, H3K4me3 and H3K27ac are found at the promoters of active genes (Heintzman et al. 2009; Creyghton et al. 2010). In contrast, H3K27me3 and H3K9me3 are associated with gene repression, and high enrichment levels for H3K4me1 and H3K27me3 or H3K9me3 are observed at poised enhancers (Rada-Iglesias et al. 2011; Zentner et al. 2011). In particular, H3K27me3 is considered the signature of Polycomb-mediated repression (Kerppola 2009), and H3K27me3 and H3K9me3 are thought to be largely mutually exclusive (Zhang et al. 2015). The inter-CNE sequences showed enrichment levels according to the genome-wide expectation with few exceptions. Indeed, we found that the regions with the highest enrichment levels for H3K4me1 and/or H3K27me3 within the inter-CNE sequences of CNE–CNE pairs in nRDD groups 1–7 had higher enrichment levels than expected by chance in 16 out of 28 tissues (empirical *P*-value < 0.05, see supplementary fig. S8, Supplementary Material online, and Materials and Methods). In contrast, although the inter-CNE sequences of CNE–CNE pairs in nRDD groups 8–10 comprised regions with higher enrichment levels for all histone modifications compared with nRDD groups 1–7, their enrichment levels were merely similar to those expected by chance based on the genomic distances between their CNEs. Alternatively, depending on the tissue, we observed that 16–41% of the CNE–CNE pairs exhibited enrichment levels for H3K27ac higher than the background signal (see Materials and Methods) at least at one CNE. Interestingly, the number of CNE–CNE pairs for which both CNEs had higher enrichment levels than the background signal in the same tissue was significantly higher than expected by chance for all tissues (odds ratios 1.8–19, *P*-values < 2.2 × 10^−^^16^, Fisher’s Exact test), and we made similar observations for H3K27me3 (odds ratios 1.2–15, *P*-values < 2.2 × 10^−16^, Fisher’s Exact test). Together with the evolutionary constraints observed for the inter-CNE sequences, these findings hint at a functional relationship between the two CNEs of a CNE–CNE pair.

**Fig. 3.**
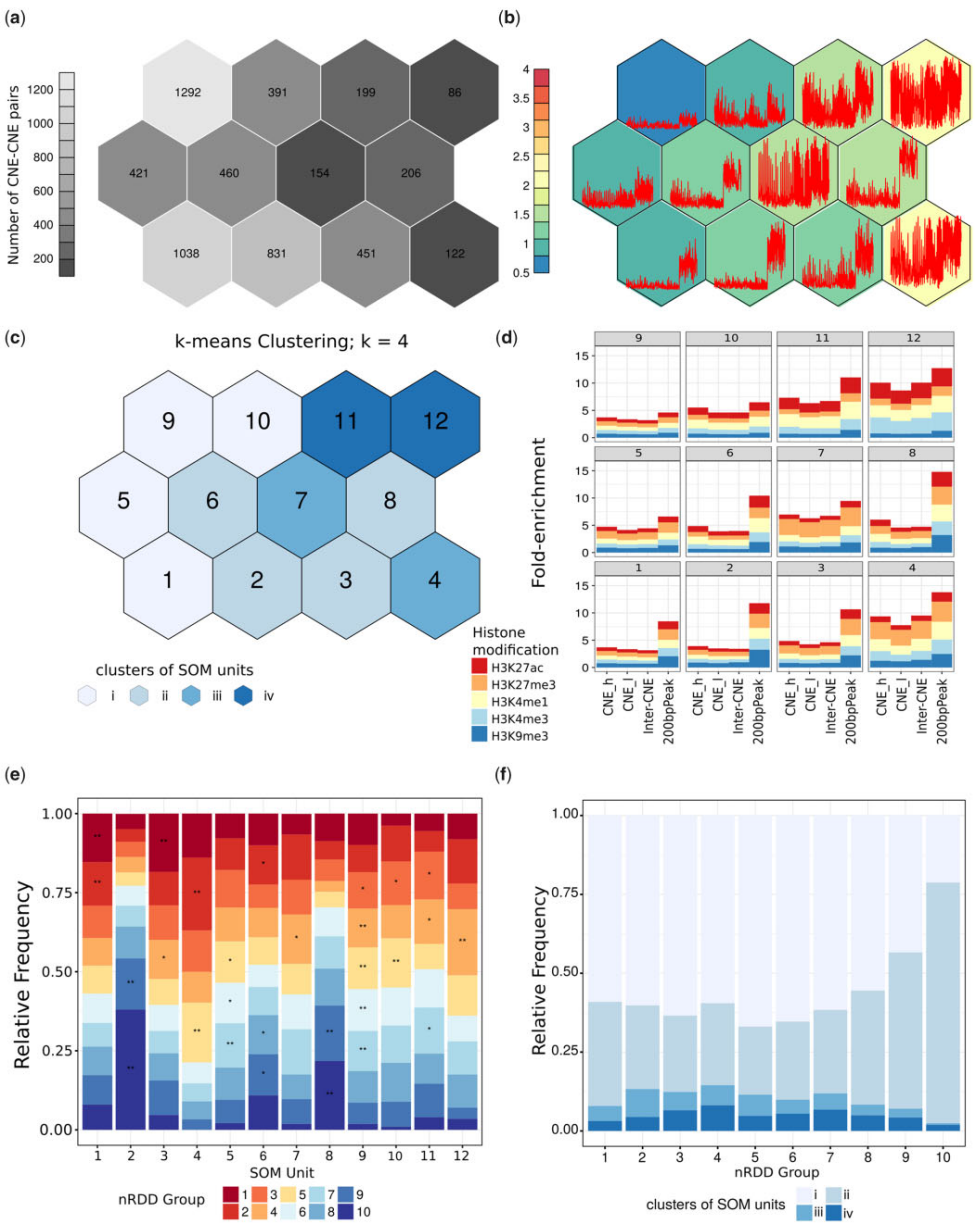
—Clustering of human CNE–CNE pairs according to their epigenetic profiles. Human mammalian conserved CNE–CNE pairs were clustered using SOMs into 12 units according to their enrichment levels (fold enrichments compared with the control experiment) for H3K27ac, H3K27me3, H3K4me1, H3K4me3, and H3K9me3 in 21 human tissues at: 1) each CNE, sorted into “CNE_h” (“high”) and “CNE_l” (“low”) according to their enrichment levels for H3K27ac (see Materials and Methods); 2) the inter-CNE sequence; and 3) the 200-bp regions with the highest enrichment levels within the inter-CNE sequences for the histone modifications under consideration. The hexagons represent SOM units. (*a*) Number of CNE–CNE pairs associated with each unit. (*b*) The background color of the hexagons represents the mean enrichment levels across all measurements for the CNE–CNE pairs associated with each unit. The red lines in the hexagons represent the means for each tissue and histone modification at each of the four regions of the CNE–CNE pairs examined in the analysis for each unit. The first two-fourths of the points correspond to the two CNEs (“h” and “l”, in that order) (1); the third fourth is the means for the inter-CNE sequences (2); the fourth fourth is the 200-bp regions with the highest enrichment levels (3). (*c*) Clusters of SOM units (see Materials and Methods). The numbering of the units is indicated in black. (*d*) Unit means across tissues and CNE–CNE pairs for each histone modification at each of the four regions of the CNE–CNE examined. (*e*) Relative frequencies of the 10 nRDD groups in the 12 SOM units and different levels of significance for the enrichment of each group in each SOM unit relative to the total number of CNE–CNE pairs: *P-value < 0.05, **FDR < 0.05. (*f*) Relative frequency of the four clusters of SOM units among the ten nRDD groups.

To identify patterns among the epigenetic profiles of the CNE–CNE pairs in different nRDD groups we applied SOMs. More precisely, we used SOMs to cluster human CNE–CNE pairs according to their enrichment levels for multiple histone modifications and tissues at the CNEs, the inter-CNE sequences, and the 200-bp-long regions with the highest enrichment levels within the latter (see Materials and Methods and figs. 3*a* and *b*). We found four clusters of SOM units (see figs. 3*a*–*d* and supplementary table S3, Supplementary Material online): i) ∼56% of the CNE–CNE pairs were associated with low enrichment levels for all histone modifications across their entire sequence, indicating either no or very low *cis*-regulatory activity (units 1, 5, 9 and 10). ii) ∼34% of the CNE–CNE pairs were characterized by low enrichment levels at the CNEs and inter-CNE sequence, except for isolated regions of moderate to high enrichment in the latter (units 2, 3, 6, and 8); in particular, the inter-CNE sequences of these CNE–CNE pairs comprised regions with relatively high enrichment levels for H3K4me1, H3K9me3, and H3K27me3, which are presumably poised enhancers. iii) ∼5% of the CNE–CNE pairs had moderate-to-high enrichment levels at the CNEs and the inter-CNE sequences (units 4 and 7), especially for H3K27me3, indicating poised enhancers. Noticeably, depending on the tissue, 25–99% (mean = 84%) of all the CNE–CNE pairs in cluster iii comprised two CNEs exhibiting higher enrichment levels for H3K27me3 than the background signal, indicating that both CNEs are poised enhancers in the same tissue. And iv) ∼5% of the CNE–CNE pairs had high enrichment levels for H3K4me1, H3K4me3, and H3K27ac across their entire sequences (units 11 and 12) and are likely to be active enhancers and promoters. Analogously to what we observed for cluster iii, depending on the tissue, 19–77% (mean = 52%) of all the CNE–CNE pairs in cluster iv comprised two CNEs featuring higher enrichment levels for H3K27ac than the background signal, indicating that both CNEs are active enhancers in the same tissue. These data strengthen the hypothesis of a functional relationship between the CNEs in the CNE–CNE pairs.

The SOMs also revealed group-specific enrichment patterns (see figs. 3*e* and *f* and Materials and Methods, all reported enrichments correspond to an FDR < 0.05 and *P*-values computed with Fisher’s exact test). Groups of CNE–CNE pairs with contracted inter-CNE distance (nRDD groups 1, 2, and 3, see supplementary table S4, Supplementary Material online) were all enriched among units of cluster i (groups 1 and 2 in unit 1 and group 3 in unit 10, with odds ratios 1.9, 1.6, and 1.5, respectively), characterized by no or very low *cis*-regulatory activity. In addition, group 1 was enriched among units of cluster ii (unit 3, odds ratio = 2.2), which comprise CNE–CNE pairs with likely poised enhancers in their inter-CNE sequences, while groups 2 and 3 were enriched among units of clusters iii (group 2 in unit 4 with odds ratio = 2.8) and iv (group 3 in units 10 and 11 with odds ratios 1.5 and 1.6, respectively), which encompass CNE–CNE pairs with poised and active enhancer activities at the CNEs, respectively. Moreover, as opposed to the expected 30%, 40% of the CNE–CNE pairs in cluster iii were CNE–CNE pairs with contracted inter-CNE sequences. These observations suggest that the CNEs in pairs with extremely contracted inter-CNE sequences have no or very low enhancer activity but may neighbor stronger *cis*-regulatory elements with poised enhancer activity in the inter-CNE sequence, while CNEs in pairs with only mildly contracted inter-CNE sequences are more likely to constitute poised and active enhancers. Groups with conserved inter-CNE distances (nRDD groups 4–7) were all mildly enriched among units of cluster i (unit 9, odds ratios 1.4–1.5), but showed the highest enrichment among units of clusters iii and iv (group 5 in unit 4 with odds ratio = 2.1 and group 4 in unit 12 with odds ratio = 2.4, respectively), which comprise CNE–CNE pairs with poised and active enhancer activities at the CNEs, respectively. Furthermore, clusters iii and iv are enriched in CNE–CNE pairs with conserved inter-CNE sequences (with odds ratios 1.2 and 1.3, respectively). Indeed, CNEs in CNE–CNE pairs with conserved inter-CNE distances, and among them, nRDD groups 4 and 7, are the most likely to constitute poised and active enhancers among all CNE–CNE pairs (see fig. 3*f*). In particular, the fact that CNEs in nRDD groups 4 and 7 are comparatively less conserved but more likely to be functional than those in nRDD groups 5 and 6 might reflect a need for small adjustments in response to other genomic changes. Finally, groups of CNE–CNE pairs with expanded inter-CNE distances (nRDD groups 8, 9, and 10) were enriched among the units of cluster ii (group 9 in units 2, 6 and 8, with odds ratios 2.0, 1.4 and 2.0, respectively, and group 10 in units 2 and 8, with odds ratios 11.0 and 2.6, respectively; group 8 is not enriched in any units), characterized by low enrichment levels at the CNEs and inter-CNE sequence, except for isolated regions of high enrichment in the latter. The relatively high enrichment levels for H3K9me3 observed for these CNE–CNE pairs are suggestive of transposon repression consistent with the high transposon densities in their inter-CNE sequences (see fig. 2*c*; Walter et al. 2016). Our findings suggest that the CNEs in pairs with expanded inter-CNE sequences have low enhancer activity; on the contrary, their inter-CNE sequences are likely to contain at least one poised and/or active enhancer, but as mentioned above, the expectation is the same as that for sequences that are under no evident constraint (see supplementary fig. S8, Supplementary Material online). Deeply conserved CNE–CNE pairs exhibited similar patterns as mammalian CNE–CNE pairs (see supplementary fig. S9, Supplementary Material online).

Overall, our data give evidence that different conservation levels of the inter-CNE distances are associated with distinct epigenetic profiles. Specifically, CNEs in pairs with conserved or mildly contracted inter-CNE sequences are the most likely to represent poised and active enhancers, whereas both extreme contractions and expansions are associated with relatively little *cis*-regulatory activity at the CNEs (see fig. 3*f*). In addition, the unexpectedly high proportions of CNE–CNE pairs in which both CNEs have the epigenetic signature of poised or active enhancers are strongly suggestive of a functional relationship between the CNEs. Together, these two results hint at a synergistic interaction between the CNEs. Finally, in contrast to contracted inter-CNE sequences, expanded inter-CNE sequences are not more likely to contain *cis*-regulatory elements than expected by chance. Hence, our findings are consistent with the entire CNE–CNE sequence acting as a regulatory unit comprising multiple cooperative *cis*-regulatory elements, which is disrupted by extreme changes in the inter-CNE distance.

### CNEs in Human and Mouse Orthologous CNE–CNE Pairs with Conserved Inter-CNE Distances Are Associated with *Cis*-Regulatory Activity

**Fig. 4.**
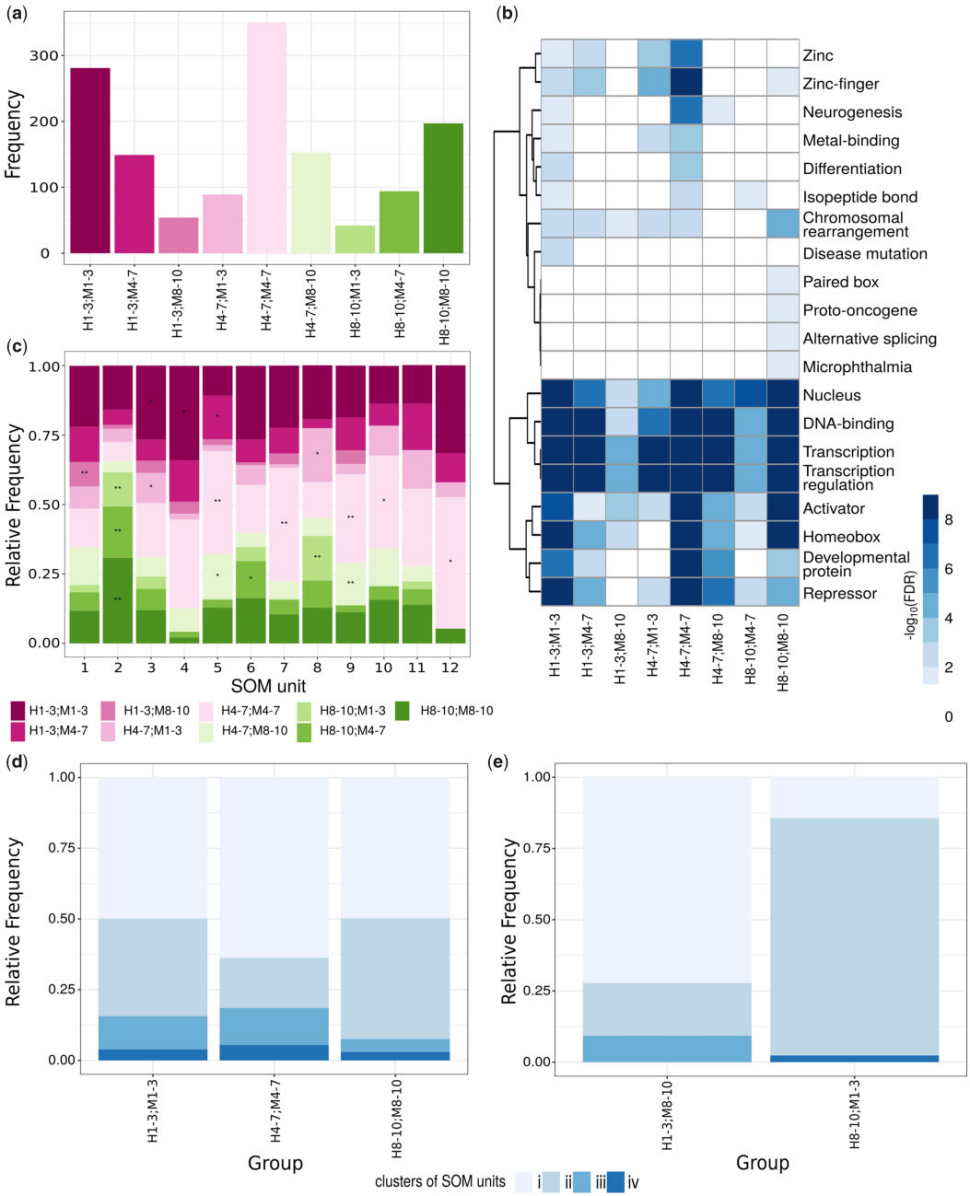
—Analysis of human and mouse AO CNE–CNE pairs. Ranges of human and mouse nRDD groups are preceded by “H” and “M,” respectively. (*a*) Number of AO CNE–CNE pairs. (*b*) Functions significantly enriched among genes in the neighborhood of AO CNE–CNE pairs. All CNE–CNE pairs in the human genome were used as background. All terms not associated with white are significant (FDR < 0.05). (*c*) Relative frequencies of the human orthologs of the AO CNE–CNE pairs in the 12 SOM units (see fig. 3). Enrichment of each category in each SOM unit was calculated relative to the total number of AO CNE–CNE pairs: *P-value < 0.05, **FDR < 0.05. (*d*) Relative frequency of the human orthologs of the AO CNE–CNE pairs with consistent contraction/expansion trends in the human and mouse genomes in the four SOM clusters (see fig. 3*c*). (*e*) Relative frequency of the AO CNE–CNE pairs with opposite contraction/expansion trends in the human and mouse genomes in the four SOM clusters.

To investigate lineage-specific changes, we performed an analogous analysis on the mouse genome. Specifically, we identified 2,084 mammalian conserved CNE–CNE pairs that exhibited similar sequence and epigenetic profiles as their human counterparts (see supplementary figs. S10 and S11 and tables S5 and S6, Supplementary Material online). Next, we compared the mammalian conserved CNE–CNE pairs in the human and mouse genomes, and identified a subset of 1,411 adjacent orthologous CNE–CNE pairs (AO CNE–CNE pairs) in the both genomes (see Materials and Methods). The vast majority of AO CNE–CNE pairs exhibited consistent contraction/expansion trends in their inter-CNE sequences in both genomes, in the sense they were either contracted, conserved, or expanded in both human and mouse (see fig. 4*a* and supplementary fig. S12, Supplementary Material online). Only, 54 AO CNE–CNE pairs represented contractions in the human (nRDD groups 1–3) but expansions in the mouse genome (nRDD groups 8–10), whereas 42 were expansions in the human (nRDD groups 8–10) but contractions in the mouse genome (nRDD groups 1–3). Also, we found that AO CNE–CNE pairs with consistent contraction/expansion trends in the human and mouse were associated with more biological processes, molecular functions, and protein domains than AO CNE–CNE pairs with opposite contraction/expansion trends (contracted inter-CNE sequences in human and expanded in mouse or vice versa, see fig. 4*b* and Materials and Methods). This supports the hypothesis that local contractions and expansions have specifically shaped the mammalian genome, whereas opposite contractions/expansions trends are, in addition to rare, mainly unspecific.

Further, we were interested in understanding whether the AO CNE–CNE pairs were associated with specific regulatory functions and how such functions compared with the remaining CNE–CNE pairs in the human and mouse genomes. For this purpose, we examined the epigenetic profiles of AO CNE–CNEs using the afore-presented SOMs (see figs. 3*a* and 4*c* and Materials and Methods, all reported enrichments correspond to a *P*-value < 0.05 computed with Fisher’s exact test). As the entire collection of CNE–CNE pairs in the human genome, a relatively large proportion of all AO CNE–CNE pairs (57%) were associated with generally low enrichment levels (cluster i, units 1, 5, 9, and 10).

The AO CNE–CNE pairs with consistent contraction/expansion trends in their inter-CNE sequences in both genomes featured a similar distribution with respect to the four SOM clusters as the entire collection of human CNE–CNE pairs (see fig. 4*d* and supplementary fig. S13*a*, Supplementary Material online). Specifically, we found that AO CNE–CNE pairs with contracted inter-CNE sequences (nRDD groups 1–3) in both genomes were enriched among the units of clusters ii (unit 3, odds ratio = 1.5, see fig. 4*c*) and iii (unit 4, odds ratio = 2.1). In addition, although the AO CNE–CNE pairs with contracted inter-CNE sequences in both genome were not significantly enriched among any of the units of cluster iv, they showed a trend toward overrepresentation (odds ratio = 1.9 in unit 12). AO CNE–CNE pairs with conserved inter-CNE sequences (nRDD groups 4–7) in both genomes were enriched among the units of clusters i (units 5, 9, and 10, odds ratios 1.9, 1.6, and 1.6), iii (unit 7, odds ratio = 2.2), and iv (unit 12, odds ratio = 2.8). Finally, AO CNE–CNE pairs with expanded inter-CNE sequences (nRDD groups 8–0) in both genomes were enriched among the units of cluster ii (unit 2, odds ratio = 3.3). Therefore, similar to the entire collection of CNE–CNE pairs in the human genome, the CNEs in pairs with conserved and contracted inter-CNE sequences in both genomes were the most likely to constitute poised and active enhancers and promoters. Compared with the entire collection of CNE–CNE pairs in the human genome, AO CNE–CNE pairs with consistent contraction/expansion trends had a higher proportion of pairs in cluster iii, with odds ratios ranging between 1.8 and 2.3 (see fig. 4*d* and supplementary fig. S13*a*, Supplementary Material online). As expected considering that most AO CNE–CNE pairs showed consistent contraction/expansion trends in their inter-CNE sequences, the same observations were made for the entire collection of AO CNE–CNE pairs (see supplementary figs. S13*a* and *b*, Supplementary Material online).

In contrast, the AO CNE–CNE pairs with opposite contraction/expansion trends only featured a similar distribution as the entire collection of human CNE–CNE pairs with respect to the SOM clusters i and ii (see fig. 4*e* and supplementary fig. S13*a*, Supplementary Material online). Specifically, we found that AO CNE–CNE pairs with opposite contraction/expansion trends were enriched among the units of clusters i (human nRDD groups 1–3 and mouse nRDD groups 8–10 in unit 1, odds ratio = 3.2) and ii (human nRDD groups 8–10 and mouse nRDD groups 1–3 in units 2 and 8, odds ratios 7.3 and 7.0, respectively). Different from the entire collection of human CNE–CNE pairs, we observed no AO CNE–CNE pairs with contracted inter-CNE sequences in the human genome and expanded inter-CNE sequences in the mouse genome in cluster iv, and no AO CNE–CNE pairs with expanded inter-CNE sequences in the human genome and contracted inter-CNE sequences in the mouse genome in cluster iii (see fig. 4*e*).

In summary, the epigenetic profiles of AO CNE–CNE pairs with different conservation levels of inter-CNE distances are consistent with our observations in the human and mouse genomes. Furthermore, among all AO CNE–CNE pairs, the CNEs of the pairs with opposite contraction/expansion trends in the human and mouse genomes represent a small minority and are the least likely to be associated with *cis*-regulatory activity, suggesting a convergent evolutionary force on both genomes. These results provide additional support for the hypothesis that CNE–CNE pairs act as functional units and that the inter-CNE distance is relevant to the *cis*-regulatory activity of the corresponding CNEs.

## Discussion

The genomic distances between highly conserved sequences (Bejerano 2004; Sandelin et al. 2004; Woolfe et al. 2004) have been shown to be more conserved than those between (protein-)coding sequences (Sun et al. 2006, 2009). Consistent with these observations, it has been hypothesized that the genomic sequence between pairs of CNEs contains functional elements and/or is structurally relevant for transcriptional regulation or other cellular processes. In contrast to Sun et al., who applied comparative genomics to describe inter-CNE distances in the human genome relative to other seven vertebrate species, we considered 23 vertebrate species and inferred the ancestral inter-CNE distances in extinct mammalian species to detect patterns of selection in the mammalian genome and, in particular, in the human genome. Specifically, we reconstructed the inter-CNE distances in the ancestral mammalian genomes using the maximum likelihood method (Felsenstein 1973) using fixed tree topology and branch lengths. A known limitation of this approach is that different genomic regions may have diverged at different rates across distinct species. Indeed, our method is underpowered to detect species-specific changes, which would be better captured by inferring both the evolutionary tree topology and corresponding branch lengths for each CNE–CNE pair. In addition, to account for global changes in genome size, and thus, expose changes in the inter-CNE distance that do not reflect the general trend of the genome, we developed the nRDD metric. We found that the nRDD values of the CNE–CNE pairs are only weakly correlated with the inter-CNE distances. Even if weak, the fact that there is a reliable association between the nRDD values and inter-CNE distances suggests that the evolutionary forces that govern local genomic contractions and expansions are constrained by the spatial context. Despite these confounding factors, the nRDD offers a valuable framework for assessing the contraction/expansion trends of inter-CNE sequences in the genome.

In order to uncover the possible causes of contraction/expansion in inter-CNE sequences of CNE–CNE pairs, we compared the transposon densities of human and mouse inter-CNE sequences with those inferred for the common mammalian ancestor. Transposons comprise ∼45% and 37.5% of the human and mouse genome, respectively (Lander et al. 2001; Waterston 2002), and have had a remarkable impact on the evolution of the mammalian genome, greatly contributing to the alteration of gene regulatory networks (Friedli and Trono 2015). Also, transposon activity has been associated with human disease, such as cancer (Chenais 2015). Except for extremely expanded inter-CNE sequences, inter-CNE sequences showed substantially lower transposon densities than expected by chance, indicating the existence of a strong evolutionary constraint on such sequences. Moreover, we present evidence that this constraint is not specific to the human or mouse genome and most likely was already present in the common mammalian ancestor. Indeed, the CNE–CNE pairs with conserved inter-CNE distances were depleted in transposon deletions and insertions in both the human (mouse) and common mammalian ancestor genomes. On the contrary, CNE–CNE pairs with contracted and expanded inter-CNE distances were mainly enriched in transposon deletions and insertions, respectively, in the human (mouse) genome. Furthermore, although LINEs compose the largest fraction of extremely expanded inter-CNE sequences, we showed that their insertions predate the last common mammalian ancestor. Rather than LINEs, the extreme expansions in inter-CNE sequences in the human (mouse) genome appear to be mainly driven by SINE insertions, which are found across most eukaryotes but have accumulated to large amounts in mammals (Richardson et al. 2015). The strong evolutionary constraint on most CNE–CNE pairs makes them strong candidates for functional noncoding sequences.

Many CNEs have been shown to have *cis*-regulatory activity (Loots et al. 2000; Woolfe et al. 2004; Pennacchio et al. 2006; Dickel et al. 2018). To determine whether CNE–CNE pairs with different conservation levels of inter-CNE distances are associated with distinct *cis*-regulatory functions, we examined the epigenetic profiles at the CNEs and inter-CNE sequences using SOMs. We found that most CNE–CNE pairs were associated with low enrichment levels for all histone modifications considered across their entire sequence. This might be at least partially explained by the relatively low number of tissues considered, and some of these sequences might actually have *cis*-regulatory activity in other tissues. Moreover, the enrichment level for H3K27ac is simply a proxy for regulatory activity and the decision of whether the histone modification is present or not relies on an arbitrary threshold. Thus, including more tissues in the analysis and setting less stringent thresholds would most likely increase the proportion of active CNEs. In fact, 53% of the CNEs in mammalian conserved CNE–CNE pairs overlap with cell type-agnostic candidate regulatory regions identified by the ENCODE Project (http://screen.encodeproject.org/, last accessed September 12, 2018; ENCODE Project Consortium 2011, 2012). More importantly, we found that the conservation level of the inter-CNE distance is informative about the *cis*-regulatory activity of the CNEs and inter-CNE sequences of a pair. Specifically, CNEs in CNE–CNE pairs with conserved and mildly contracted inter-CNE distances are more likely to be poised or active enhancers compared with CNEs in pairs with either extremely contracted or expanded inter-CNE sequences, and are good candidates for transgenic enhancer assays, whereas both extreme contractions and expansions are associated with relatively little *cis*-regulatory activity at the CNEs. Moreover, we observed that often the two CNEs in a pair show very similar epigenetic profiles, at a rate that is much higher than expected by chance. Taken together, these results suggest that pairs of functional adjacent CNEs act as regulatory units, with considerable functional interactions between the CNEs. Further, our data are consistent with extreme inter-CNE contractions and expansions largely disrupting such interactions and lead to loss of function at the CNEs, while mild inter-CNE contractions often result in regulatory innovation. Further, our data indicate that among CNE–CNE pairs with relatively well conserved inter-CNE distances, CNEs in those with slight changes in their inter-CNE distances are associated with the greatest likelihood of *cis*-regulatory activity, perhaps reflecting a strategy for fine-tuning in response to other genomic changes. The fact that our results are similar for both mammalian and deeply conserved CNE–CNE pairs, and even more evident for CNE–CNE pairs under stronger evolutionary constraints, suggests that this is a general evolutionary trend.

Our analyses rely on a series of parameter values. PhastCons elements represent genomic sequences with a wide range of evolutionary conservation levels (Siepel 2005) and have extensively been used to study *cis*-regulatory activity (Visel et al. 2007; Gittelman et al. 2015; Emera et al. 2016). We did not consider elements shorter than 100 bp or longer than 1,000 bp to reflect the size of a typical *cis*-regulatory element (Arnosti 2003). We applied an RBH search strategy to identify the orthologs of 5,657 pairs of CNEs in 23 vertebrate genomes. As a compromise between speed and sensitivity, we used BLAT (Kent 2002). Given that BLAT is optimized for finding very similar sequences, it is expected to miss some true BRHs, especially when run between the least similar genomes (Ward and Moreno-Hagelsieb 2014). In addition, the identification of orthologous CNEs and AO CNE–CNE pairs relies highly on assembly quality. Thus, missing sequences in an assembly may lead to missing orthologous relationships. This is also partially reflected in the deviation from 0 of the nRDD distributions (see fig. 1*c*), although the effect is confounded by the extreme changes in genome size (see supplementary table S1, Supplementary Material online). Other parameters, such as the number of nRDD groups and SOM units, are less sensitive and mainly affect the resolution of the analysis (data not shown). Last, but not least, we focused on pairs of CNEs. However, the properties we observed might result from restrictions imposed on higher-order arrangements of CNEs. Indeed, CNEs have been shown to often occur in clusters (Woolfe et al. 2004; Ovcharenko 2005; Takahashi and Saitou 2012). In turn, such clusters are overrepresented around genes involved in transcription, development, and the nervous system and coincide with topologically associating domains (Harmston et al. 2017) (PMID: 28874668), and there is evidence supporting the hypothesis that their relative location with respect to their target is relevant to gene regulation (Babarinde and Saitou 2016). Although disagreement exists, epistatic interactions are believed to play a substantial role in the genetic basis of complex disease (Ward and Kellis 2012). Nevertheless, testing for all possible interactions is unfeasible. Hence, detecting epistasis in genome-wide association studies is an area of intense theoretical interest (Niel et al. 2015). With genome-wide association studies continuing to expand the catalog of noncoding variants associated with human disease and large epigenome mapping consortia generating vast amounts of data, the development of novel approaches to prioritize candidate causal variants becomes increasingly important. Our data reveal a subset of candidate epistatic or cooperative interactions that warrant experimental investigation to improve our understanding of the genetic architecture of human disease.

## Supplementary Material

Supplementary data are available at *Genome Biology and Evolution* online.

## Supplementary Material

Supplementary Data
