# Supplementary material for: Pairs of Adjacent Conserved Noncoding Elements Separated by Conserved Genomic Distances Act as Cis-Regulatory Units
Source: Genome Biol Evol. 2018 Sep 3;10(9):2535–50. doi: 10.1093/gbe/evy196 (PMC6161761; doi:10.1093/gbe/evy196)
Supplement: Supplementary Data [file evy196_supp.zip › LiLifei_SupplementaryMaterials.pdf]

Supplementary Material

Pairs of adjacent conserved non-coding elements separated by conserved genomic distances act as *cis*-regulatory units

Lifei Li, Nicolai K. H. Barth, Eva Hirth and Leila Taher*

Division of Bioinformatics, Department of Biology, Friedrich-Alexander-Universität Erlangen-Nürnberg, Erlangen, Germany.

* Correspondence and requests for materials should be addressed to L.T. (email: leila.taher@fau.de)

Table of Contents

Supplementary Figures 3
Figure S1. Phylogenic tree for 23 vertebrate species..3
Figure S2. Comparison between human and mouse SOM units..4
Figure S3. Distance between pairs of adjacent CNEs in the human genome. 5
Figure S4. Inter-CNE distances in modern mammalian genomes compared to the inferred inter-CNE distance in the genome of the common mammalian ancestor. 6
Figure S5. nRDD and inter-CNE distance between mammalian conserved pairs of adjacent CNEs are weakly correlated. 7
Figure S6. Mean transposon density for inter-CNE sequences of deeply conserved CNE-CNE pairs in the human genome. 8
Figure S7. Relative transposon difference (nRTD). 9
Figure S8. Epigenetic profiles of mammalian conserved CNE-CNE pairs in the human genome in different nRDD groups, compared to the genome-wide expectation. 10
Figure S9. Epigenetic profiles of deeply conserved CNE-CNE pairs in the human genome in different nRDD groups, compared to the genome-wide expectation. 11
Figure S10. Sequence properties of mammalian conserved CNE-CNE pairs in the mouse genome in different nRDD groups. 12
Figure S11. Clustering of mouse CNE-CNE pairs according to their epigenetic profiles. 13
Figure S12. Consistent changes in the inter-CNE distances of human and mouse adjacent orthologous CNE-CNE pairs (“AO CNE-CNE pairs”). 15
Figure S13. Relative frequency of the four clusters of SOM units. 16
Supplementary Tables 17
Table S1. Genome size for 23 species (Ensembl “Golden Path”). 17
Table S3. Epigenetic profiles of clusters of SOM units for mammalian conserved CNE-CNE pairs in the human genome (see fig. 3). 18
Table S4. Distribution of nRDD groups across SOM units and clusters for mammalian conserved CNE-CNE pairs in the human genome. 19
Table S5. Epigenetic profiles of clusters of SOM units for mammalian conserved CNE-CNE pairs in the mouse genome (see Supplementary fig. S11). 20
Table S6. Distribution of nRDD groups across SOM units and clusters for mammalian conserved CNE-CNE pairs in the mouse genome. 21

Supplementary Figures

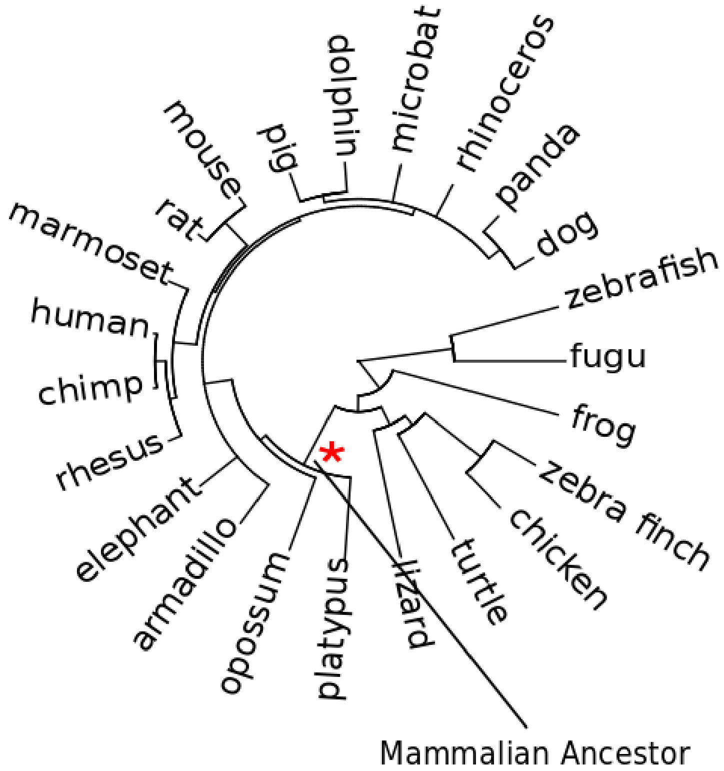

Figure S1. Phylogenic tree for 23 vertebrate species.

The last common mammalian ancestor used for the inference is denoted by a star.

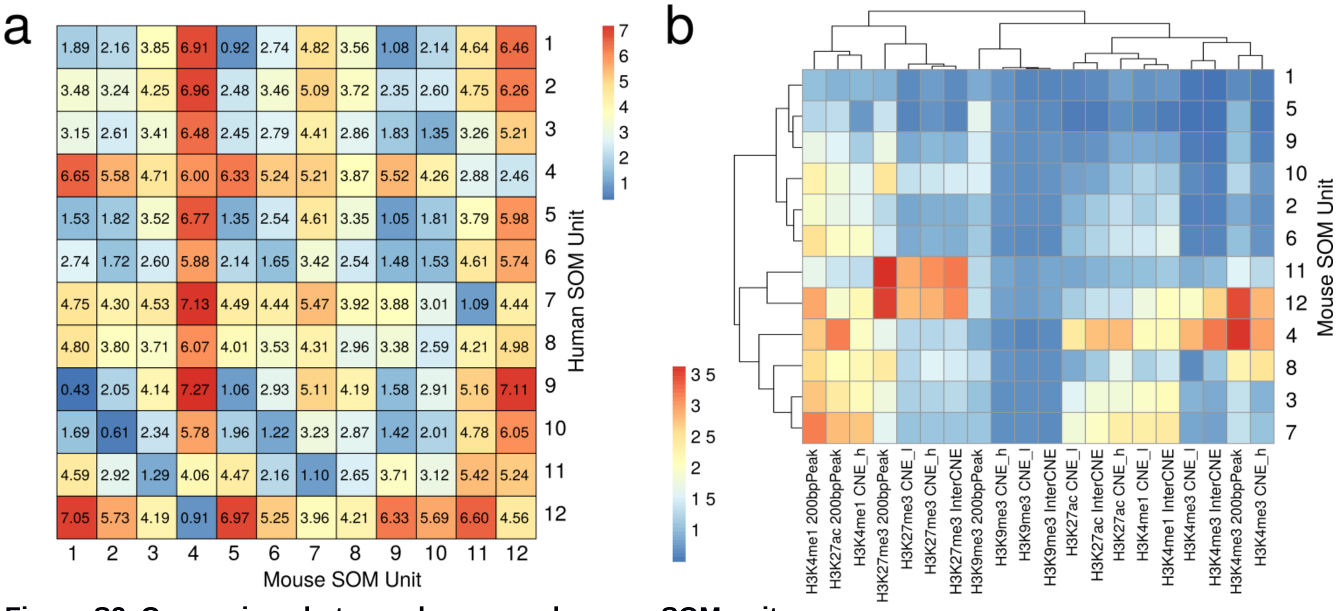

Figure S2. Comparison between human and mouse SOM units.

(a) Euclidean distance of pairwise SOM units between vectors consisting of human and mouse mean enrichment levels for histone modifications H3K4me1, H3K4me3, H3K27ac, H3K27me3 and H3K9me3 across all tissues for each histone modification and each tissue at: (i) two 300bp-long sequences, each centered at each of the CNEs (CNE_h and CNE_l, see Figs. 3 and S11); (ii) the inter-CNE sequence (interCNE); and (iii) the 200bp-long regions within the inter-CNE sequence with the highest mean enrichment levels for the histone modification under consideration (200bpPeak). The x- axis and y-axis represent the 12 mouse and human SOM units, respectively. (b) Heatmap and dendrogram exhibit the hierarchical clustering (complete linkage based on the Euclidean distance) for the 12 mouse SOM units. The x-axis indicates the histone modifications and regions within the CNE-CNE pairs (see a. for details).

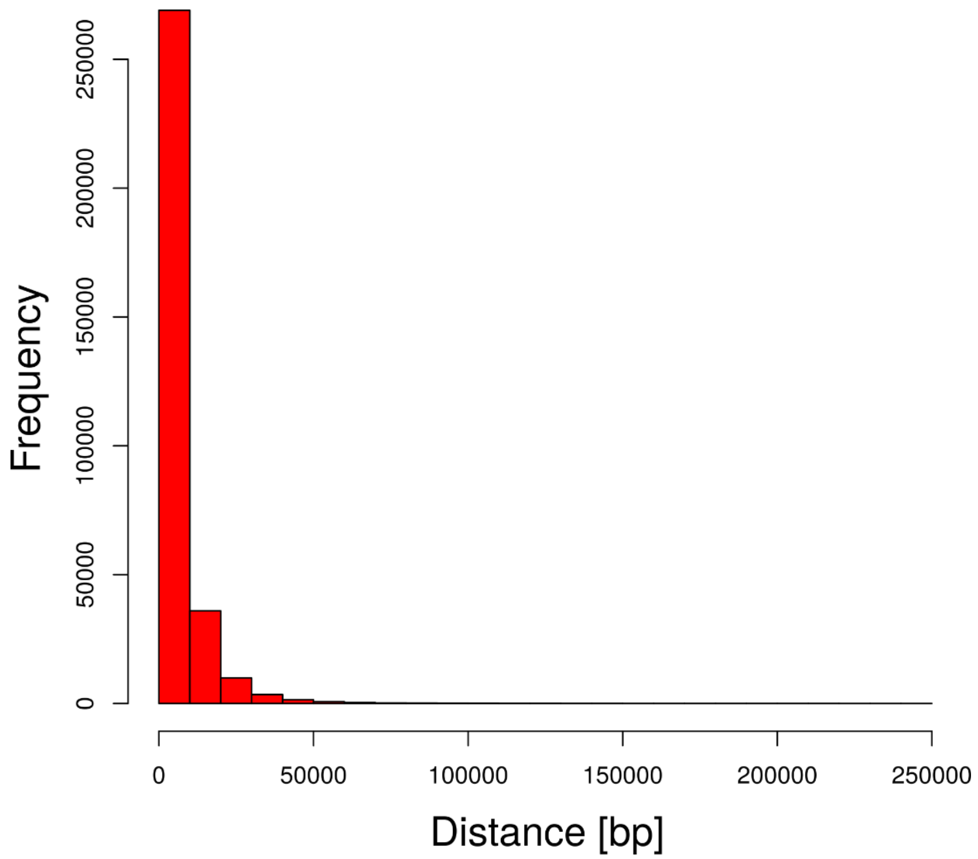

Figure S3. Distance between pairs of adjacent CNEs in the human genome.

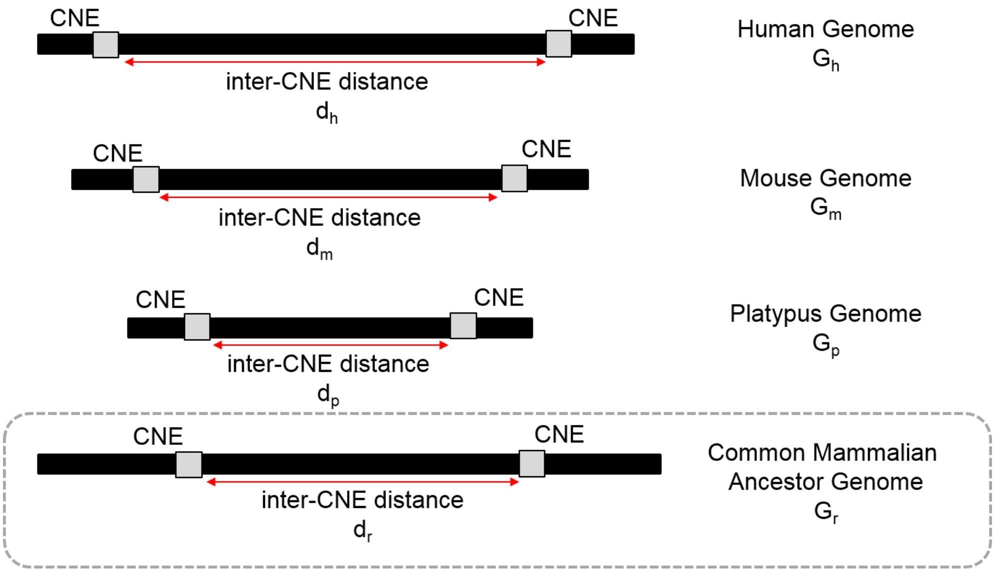

Figure S4. Inter-CNE distances in modern mammalian genomes compared to the inferred inter-CNE distance in the genome of the common mammalian ancestor.

The thick black lines represent three modern mammalian genomes (human, mouse and platypus), as well as that of the common mammalian ancestor, which serves as reference for the analysis. Their sizes are indicated by G_h (human), G_m (mouse), G_p (platypus), and G_r (reference). The gray squares correspond to CNEs. The inter-CNE distance (d_h, d_m, d_p and d_r) is the distance (in bp) between two CNEs in the genome of the species under consideration. The genome size-normalized distance is the inter-CNE distance divided by the genome size. Genome size-normalized distances are compared to the genome-size normalized distance inferred using a maximum likelihood method for the common mammalian ancestor (enclosed in the dotted box) to identify relative contractions or expansions.

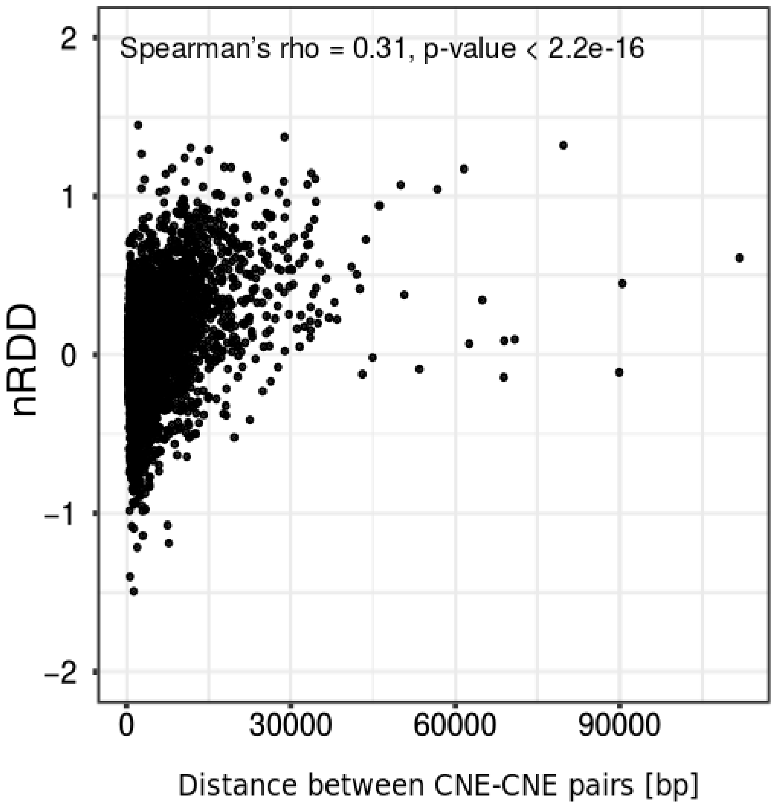

Figure S5. nRDD and inter-CNE distance between mammalian conserved pairs of adjacent CNEs are weakly correlated.

nRDD value (y-axis) as a function of the inter-CNE distance (x- axis), shown together with linear regression (blue line), confidence interval (shaded area) and Pearson's r.

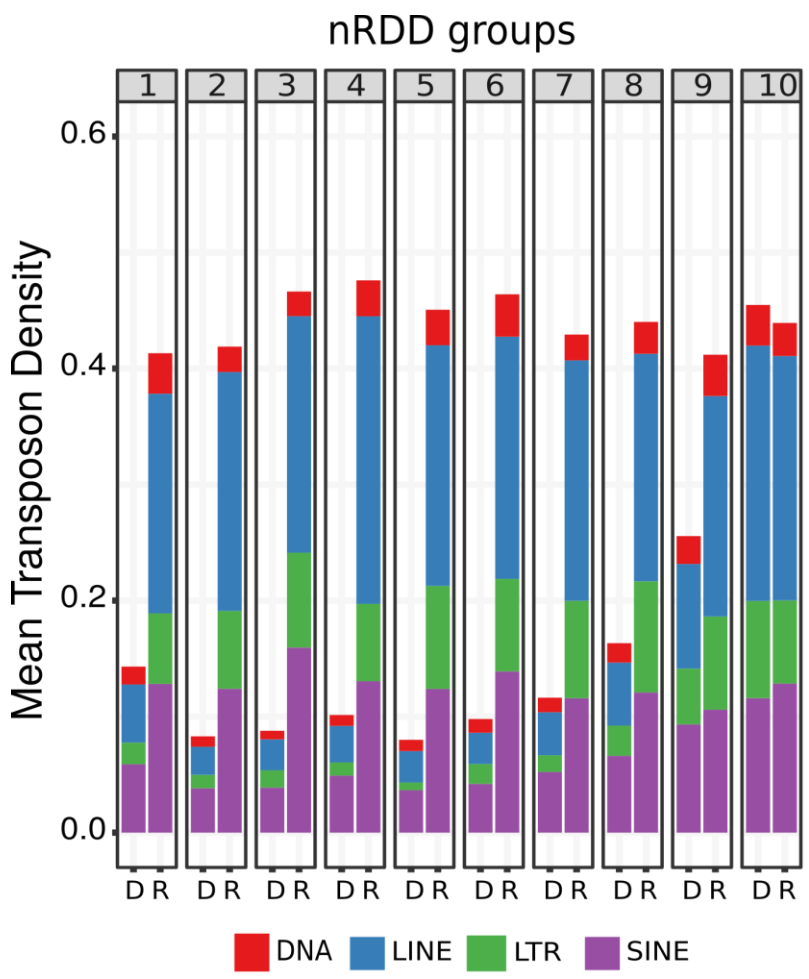

Figure S6. Mean transposon density for inter-CNE sequences of deeply conserved CNE-CNE pairs in the human genome.

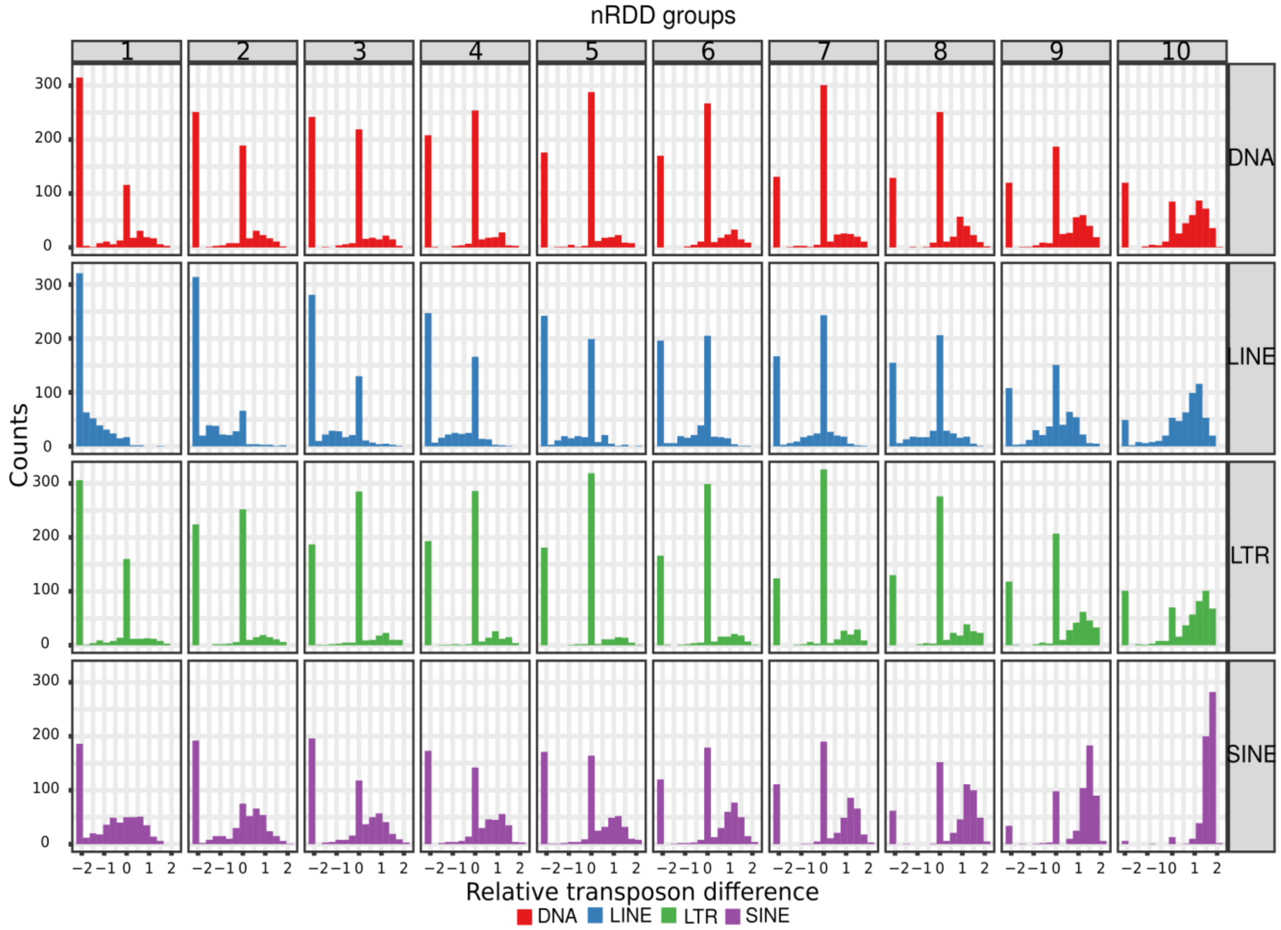

Figure S7. Relative transposon difference (nRTD).

Relative transposon difference for inter-CNE sequences of mammalian conserved CNE-CNE pairs in the human genome compared to the last common mammalian ancestor for the four main transposon families: DNA (red), LTRs (green) LINEs (blue) and SINEs (purple). On the x-axis, values from -2 to 0 represent deletions, values from 0 to 2 indicate insertions, and 0 means no deletions or insertions.

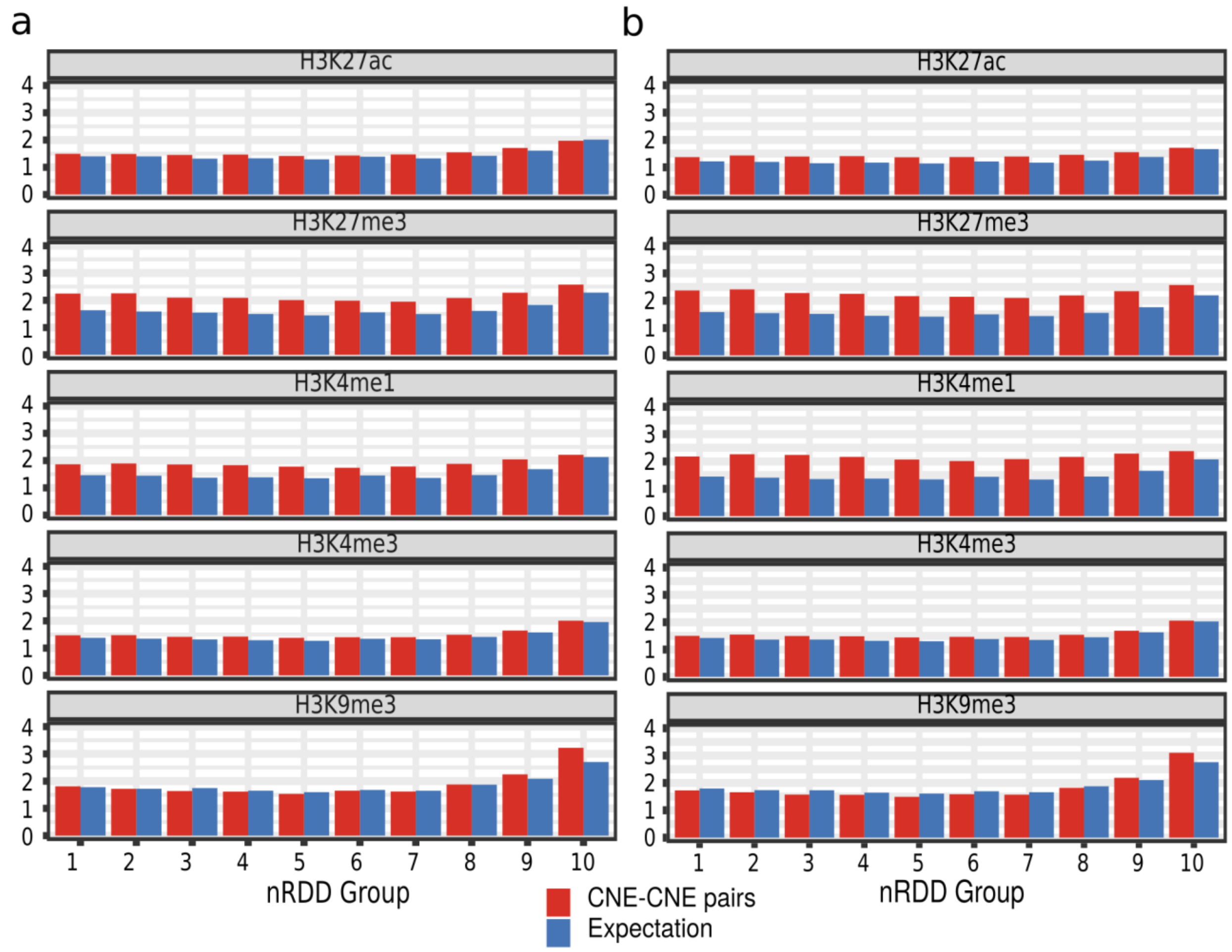

Figure S8. Epigenetic profiles of mammalian conserved CNE-CNE pairs in the human genome in different nRDD groups, compared to the genome-wide expectation.

Mean enrichment levels (fold-enrichments compared to the control experiment) for H4K27ac, H3K27me3, H3K4me1, H3K4me3 and H3K9me3 for the 200bp-regions within the inter-CNE sequences with the highest enrichment levels for the histone modifications under consideration for the CNE-CNE pairs (a) across all tissues and (b) only in the fetal and brain tissues. Each nRDD group presents in (red), compared to the mean enrichment of random genomic sequences of the same length (blue).

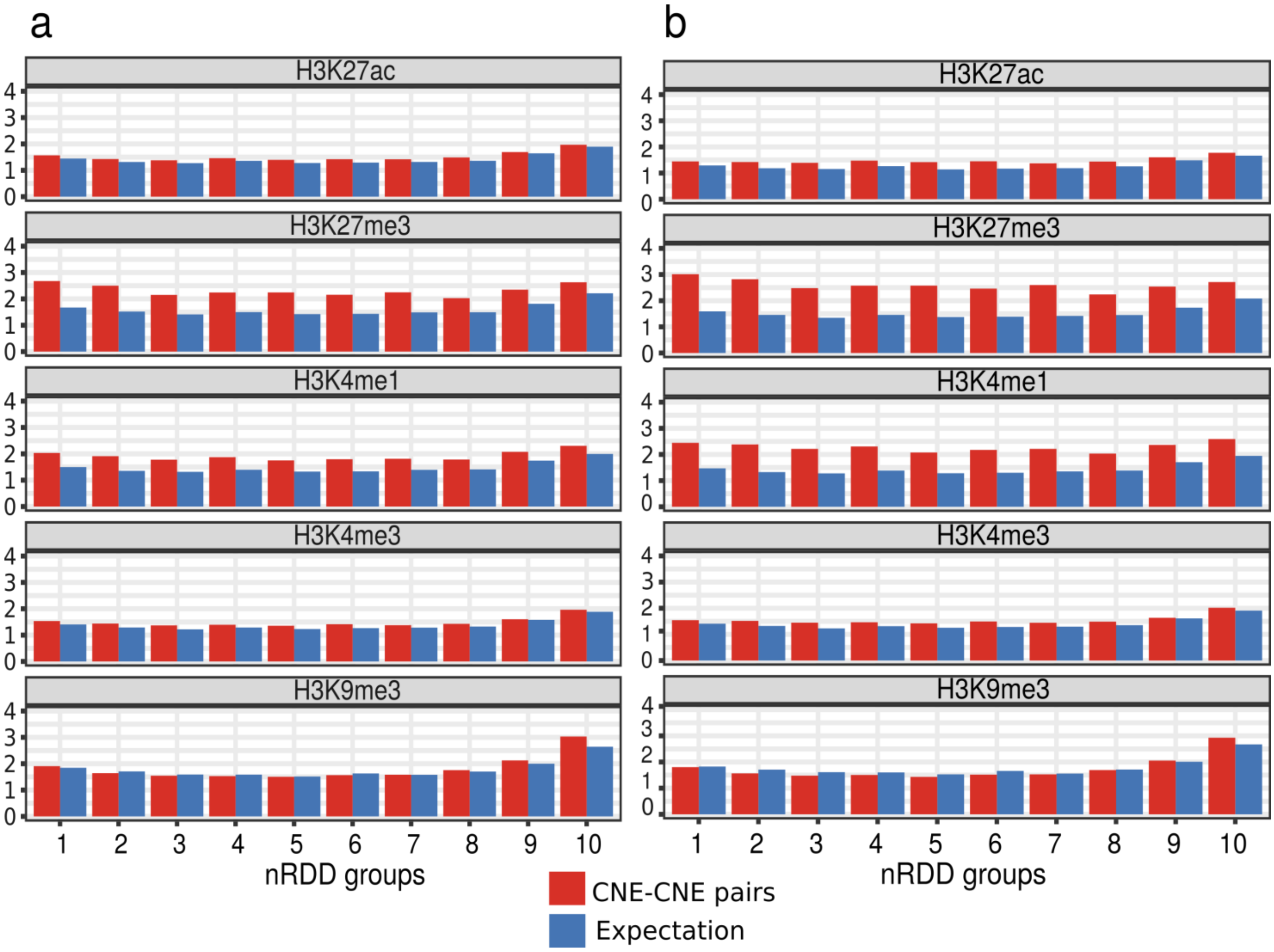

Figure S9. Epigenetic profiles of deeply conserved CNE-CNE pairs in the human genome in different nRDD groups, compared to the genome-wide expectation.

Mean enrichment levels (fold-enrichments compared to the control experiment) for H4K27ac, H3K27me3, H3K4me1, H3K4me3 and H3K9me3 for the 200bp-regions within the inter-CNE sequences with the highest enrichment levels for the histone modifications under consideration for the CNE-CNE pairs (a) across all tissues and (b) only in the fetal and brain tissues. Each nRDD group presents in (red), compared to the mean enrichment of random genomic sequences of the same length (blue).

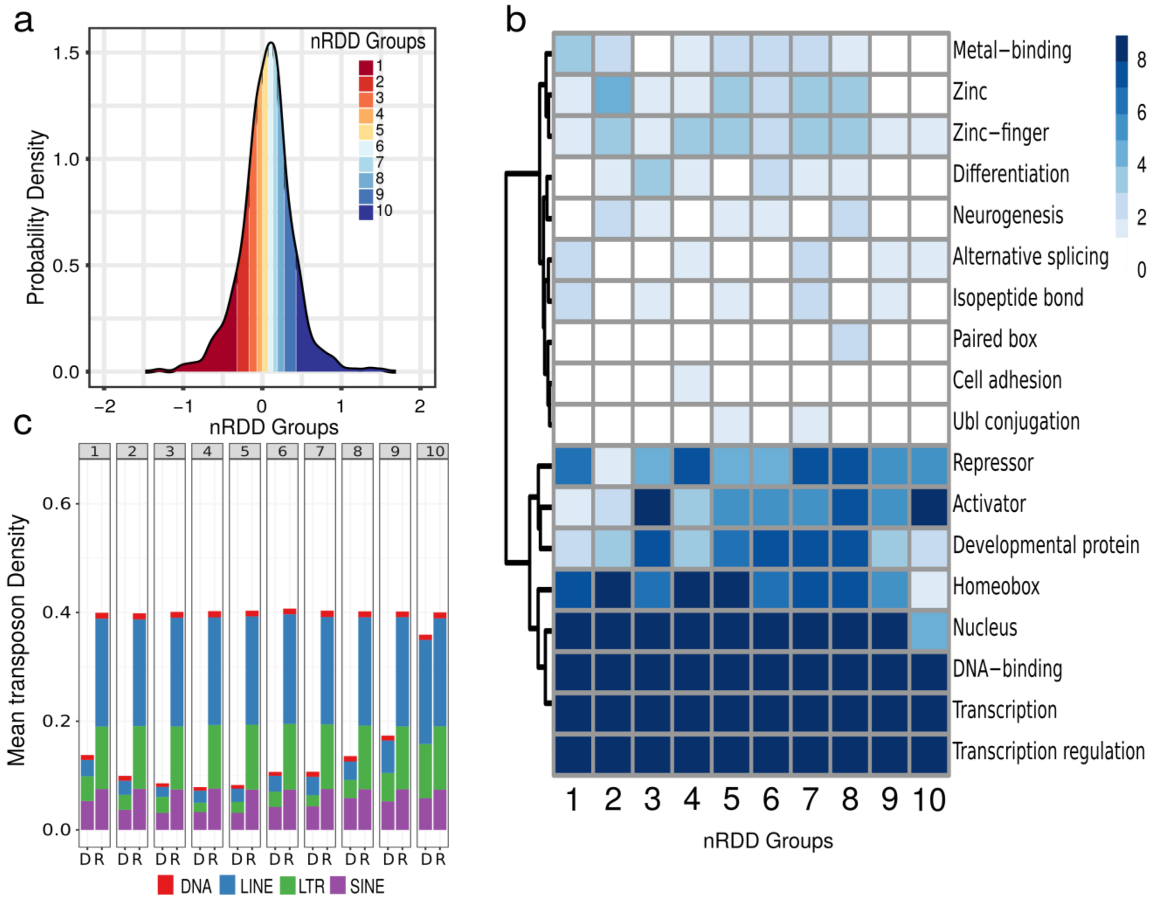

Figure S10. Sequence properties of mammalian conserved CNE-CNE pairs in the mouse genome in different nRDD groups.

(a) Distribution of nRDD values for mammalian conserved CNE-CNE pairs in the mouse genome. Ten equal-sized groups (deciles) defined according to increasing nRDD values are denoted with different colors. (b) For any given square, the color illustrates the significance ($-\log_{10}$ (FDR)) of the association between each group (x axis) and functional annotation, computed based on the annotation of the nearest gene to each CNE-CNE pair. FDR≥0.05 are represented in white. (c) Mean transposon density for the inter-CNE sequences of the CNE-CNE pairs in each group ("D") for the four main transposon families: DNA (red), LTRs (green), LINEs (blue) and SINEs (purple), compared to the genome-wide expectation according to their lengths ("R"). Except for group 10, inter-CNE sequences of CNE-CNE pairs in all groups exhibited significantly lower transposon densities than expected by chance.

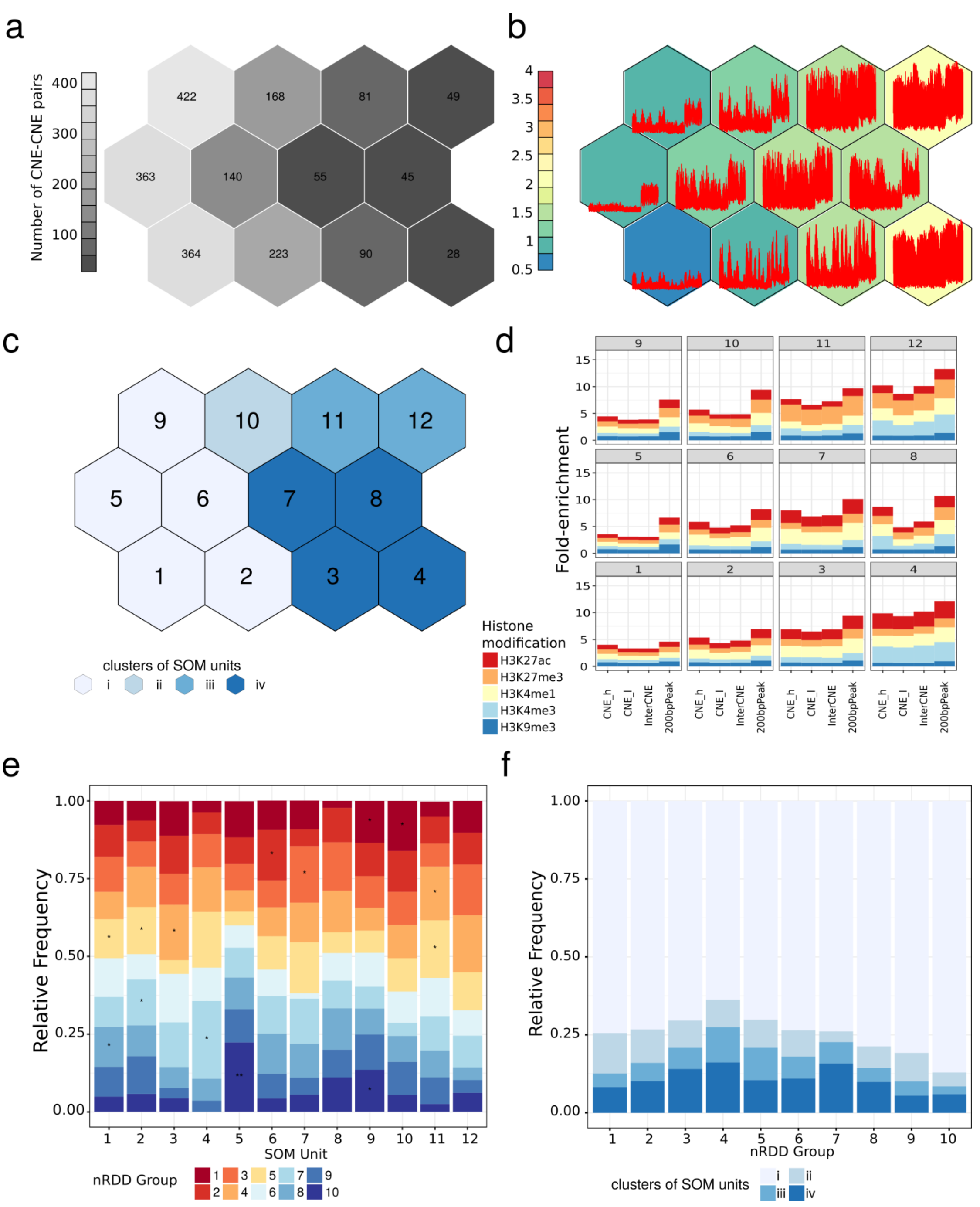

Figure S11. Clustering of mouse CNE-CNE pairs according to their epigenetic profiles.

Human mammalian conserved CNE-CNE pairs were clustered using self-organizing maps (SOMs) into 12 units according to their enrichment levels (fold-enrichments compared to the control experiment) for H4K27ac, H3K27me3, H3K4me1, H3K4me3 and H3K9me3 in 21 human tissues at: i) each CNE, sorted into “CNE_h” (“high”) and “CNE_l” (“low”) according to their enrichment levels for H3K27ac (see Methods); ii) the inter-CNE-sequence; and iii) the 200bp-regions with the highest enrichment levels within the inter-CNE sequences for the histone modifications under consideration. The hexagons represent SOM units. (a) Number of CNE-CNE pairs associated with each unit. (b) The background color of the hexagons represents the mean enrichment levels across all measurements for the CNE-CNE pairs associated with each unit. The red lines in the hexagons represent the means for each tissue and histone modification at each of the four regions of the CNE-CNE pairs examined in the analysis for each unit. The first two fourths of the points correspond to the two CNEs (“h” and “l”, in that order, i); the third fourth is the means for the inter-CNE sequences (ii); the fourth fourth is the 200-bp region with the highest enrichment levels (iii). (c) Clusters of SOM units (see Methods) The numbering of the units are indicated in black. (d) Unit means across tissues

and CNE-CNE pairs for each histone modification at each of the four regions of the CNE-CNE examined. (e) Relative frequencies of the 10 nRDD groups in the 12 SOM units and different levels of significance for the enrichment of each group in each SOM unit relative to the total number of CNE-CNE pairs: * P-value<0.05, ** FDR <0.05. (f) Relative frequency of the four clusters of SOM units among the 10 nRDD groups.

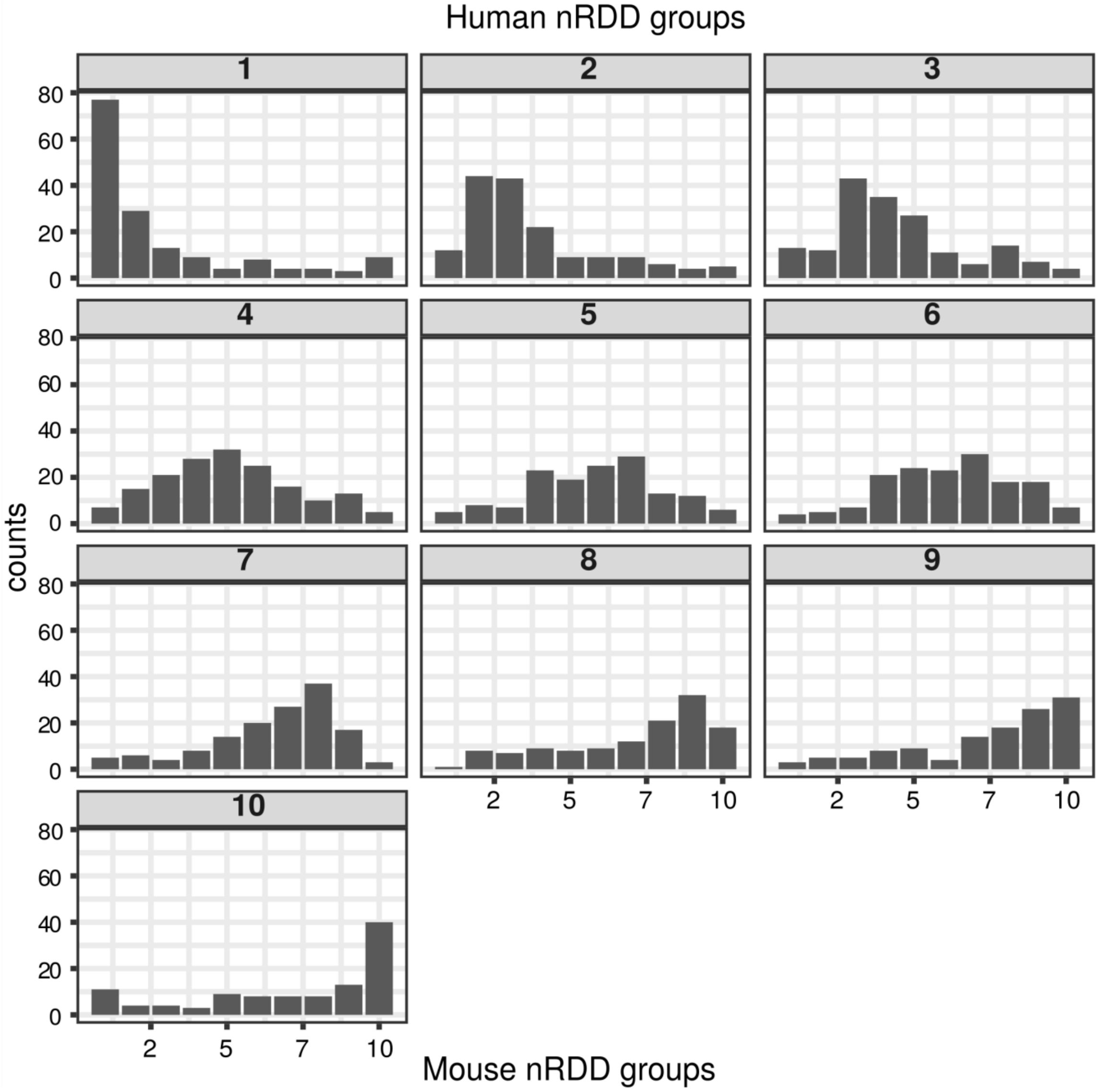

Figure S12. Consistent changes in the inter-CNE distances of human and mouse adjacent orthologous CNE-CNE pairs ("AO CNE-CNE pairs").

Each of the ten panels corresponds to a human nRDD group. The histograms show the number of CNE-CNE pairs (y-axis) in each mouse nRDD groups (x-axis). For instance, 77/160 (~48%) of the CNE-CNE pairs in human nRDD group 1 are also in mouse nRDD group 1, while 40/108 (~37%) of the CNE-CNE pairs in human nRDD group 10 are also in mouse nRDD group 10.

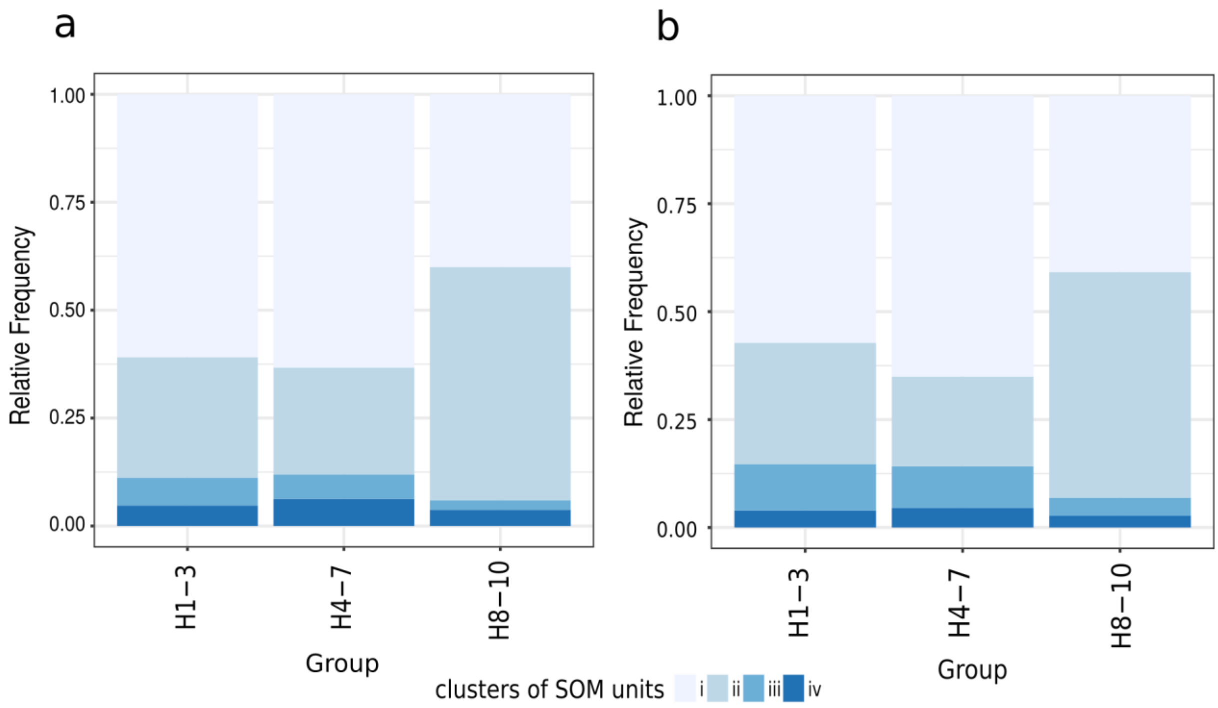

Figure S13. Relative frequency of the four clusters of SOM units.

(a) Mammalian CNE-CNE pairs; (b) AO CNE-CNE pairs with contracted inter-CNEs distance (H1-3), conserved inter-CNEs distance (H4-7), and expanded inter-CNEs distance (H8-10) in the human genome.

Supplementary Tables

Table S1. Genome size for 23 species (Ensembl "Golden Path").

Species	Genome size [bp]
Human	3,098,825,702
chimp	3,309,577,922
Marmoset	2,914,958,544
Mouse	2,730,871,774
Dog	2,410,976,875
Armadillo	3,631,522,711
Elephant	3,196,760,833
Opossum	3,605,631,728
Platypus	2,073,148,626
Chicken	1,046,932,099
Zebra finch	1,233,186,341
Turtle	2,202,483,752
Lizard	1,799,143,587
Frog	1,511,735,326
Zebrafish	1,371,709,383
Fugu	393,312,790
Rat	2,870,184,193
Rhesus	3,097,179,960
Microbat	2,034,575,300
Panda	2,299,509,015
Pig	2,808,525,991
Dolphin	2,521,923,936
Rhinoceros	2,464,350,348

Table S3. Epigenetic profiles of clusters of SOM units for mammalian conserved CNE-CNE pairs in the human genome (see fig. 3).

SOM unit cluster	SOM units	CNE-CNE Region				Histone modification associations
		CNE_h	CNE_l	Inter-CNE	200bpPeak	
i	1,5,9,10	Low	Low	Low	Low to moderate	-
ii	2,3,6,8	Low	Low	Low	Moderate to high	H3K4me1, H3K9me3, H3K27me3
iii	4,7	Moderate to high	Moderate to high	Moderate to high	Moderate to high	H3K27me3
iv	11,12	High	High	High	High	H3K4me1, H3K3me3, H3K27ac

Table S4. Distribution of nRDD groups across SOM units and clusters for mammalian conserved CNE-CNE pairs in the human genome.

Trend	nRDD Group	Enrichment	
		SOM units	SOM clusters
**contracted inter-CNE sequences**	1	1,3	i,ii
	2	1,4	i,iii
	3	10,11	i,iv
**conserved inter-CNE sequences**	4	9,12	i,iv
	5	4,9	iii,i
	6	9	i
	7	9	i
**Expanded inter-CNE sequences**	8	-	-
	9	2,6,8	ii
	10	2,8	ii

Table S5. Epigenetic profiles of clusters of SOM units for mammalian conserved CNE-CNE pairs in the mouse genome (see Supplementary fig. S11).

SOM unit cluster	SOM units	CNE-CNE Region				Histone modification associations
		CNE_h	CNE_l	Inter-CNE	200bpPeak	
**i**	1,2,5,6,9	Low	Low	Low	Low to moderate	-
**ii**	10	Low	Low	Low	Moderate	H3K4me1, H3K9me3, H3K27me3
**iii**	11,12	High	High	High	High	H3K27me3
**iv**	3,4,7,8	Moderate to high	Moderate to high	Moderate to high	High	H3K4me1, H3K3me3, H3K27ac

Table S6. Distribution of nRDD groups across SOM units and clusters for mammalian conserved CNE-CNE pairs in the mouse genome.

Trend	nRDD Group	Enrichment	
		SOM units	SOM clusters
contracted inter-CNE sequence	1	9,10	i,ii
	2	6	i
	3	7	iv
conserved inter-CNE sequence	4	3,11	iv,iii
	5	1,2,11	i,iii
	6	-	-
	7	2,4	i,iv
Expanded inter-CNE sequence	8	1	i
	9	-	-
	10	5,9	i
